# Supplementary material for: Impact of COVID-19 on healthcare utilization, cases, and deaths of citizens and displaced Venezuelans in Colombia: Complementary comprehensive and safety-net systems under Colombia’s constitutional commitment
Source: PLoS One. 2023 Mar 28;18(3):e0282786. doi: 10.1371/journal.pone.0282786 (PMC10047542; doi:10.1371/journal.pone.0282786)
Supplement: S1 File — (PDF) [file pone.0282786.s001.pdf]

SUPPORTING INFORMATION S1

List of 60 municipalities included

Supplement to

Impact of COVID-19 on healthcare utilization, cases, and deaths of citizens and displaced Venezuelans in Colombia: Complementary components of safety net and insurance systems under a constitutional commitment

In PLOS ONE 2023

by

Donald S. Shepard[1]

Adelaida Boada;[2]

Douglas Newball-Ramirez[2].

Anna G Sombrio[1]

Carlos William Rincon Perez[2]

Priya Agarwal-Harding[1]

Jamie S Jason[1]

Arturo Harker Roa[2]

Diana M. Bowser[1]

[1]The Heller School of Social Policy and Management, Brandeis University, Waltham, MA USA; [2] School of Government, Universidad de los Andes, Bogotá, Colombia

*Corresponding author: Donald S. Shepard, PhD, The Heller School for Social Policy and Management, MS035, Brandeis University, Waltham, Massachusetts 02454-9110, USA; email: shepard@brandeis.edu; Tel: +1-617-584-6664, ORCID: 0000-0003-2187-0593

February 28, 2023

Table S1.1 lists the 60 municipalities included in the study . They were selected and ranked based on the number of Venezuelan residents in 2020 according to the Colombian census.

Table S1.1. Census of Venezuelans in Colombia by municipality.
Source: Colombia Migration
CENSO VENEZOLANOS EN COLOMBIA POR MUNICIPIO
FUENTE: MIGRACIÒN COLOMBIA

Dept. code	Department	Name of municipality	2019	2020	Rank
Código_ Dept	Departamento	Nom_Municipio	Venezolanos (Venezuelans		Rango (2020)
11	Bogotá D.C.	Bogotá, D.C.	352,431	337,594	1
08	Atlántico	Barranquilla	99,251	96,360	2
54	Norte de Santander	San José De Cúcuta	104,981	96,133	3
05	Antioquia	Medellín	85,062	89,187	4
76	Valle del Cauca	Cali	62,414	59,343	5
13	Bolívar	Cartagena de Indias	52,486	51,171	6
44	La Guajira	Maicao	55,222	51,087	7
44	La Guajira	Riohacha	54,536	46,782	8
47	Magdalena	Santa Marta	43,129	41,311	9
68	Santander	Bucaramanga	40,940	40,764	10
54	Norte de Santander	Villa del Rosario	39,709	36,327	11
20	Cesar	Valledupar	37,751	34,955	12
08	Atlántico	Soledad	31,991	29,959	13
25	Cundinamarca	Soacha	26,140	26,245	14
68	Santander	Floridablanca	16,694	24,455	15
81	Arauca	Arauca	23,158	21,963	16
85	Casanare	Yopal	15,475	16,158	17
05	Antioquia	Bello	13,716	13,905	18
47	Magdalena	Ciénaga	14,648	13,715	19
66	Risaralda	Pereira	13,807	13,598	20
81	Arauca	Saravena	12,770	11,994	21
44	La Guajira	Fonseca	13,335	11,754	22
44	La Guajira	San Juan del Cesar	13,822	11,399	23
70	Sucre	Sincelejo	12,044	11,327	24
44	La Guajira	Uribia	9,343	9,087	25
54	Norte de Santander	Tibú	8,994	8,659	26
25	Cundinamarca	Chía	9,074	8,511	27
05	Antioquia	Rionegro	7,388	8,411	28
54	Norte de Santander	Pamplona	8,245	8,283	29

Table S1.1. Census of Venezuelans in Colombia by municipality (continued)					
**Dept. code**	**Department**	**Name of municipality**	**2019**	**2020**	**Rank**
**Código_ Dept**	**Departamento**	**Nom_Municipio**	**Venezolanos (Venezuelans**		**Rango (2020)**
68	Santander	Piedecuesta	7,435	8,016	30
05	Antioquia	Itagüí	7,856	7,790	31
47	Magdalena	Fundación	8,129	7,379	32
05	Antioquia	Envigado	6,991	7,331	33
54	Norte de Santander	Los Patios	7,529	7,190	34
13	Bolívar	Magangué	6,411	7,138	35
76	Valle del Cauca	Jamundí	6,342	6,827	36
63	Quindío	Armenia	6,880	6,796	37
25	Cundinamarca	Facatativá	6,075	6,673	38
68	Santander	Barrancabermeja	6,085	6,271	39
52	Nariño	Pasto	4,608	6,269	40
94	Guainía	Inírida	6,317	5,933	41
08	Atlántico	Puerto Colombia	6,682	5,899	42
23	Córdoba	Montería	6,001	5,748	43
81	Arauca	Arauquita	5,996	5,740	44
47	Magdalena	El Banco	5,954	5,634	45
25	Cundinamarca	Zipaquirá	5,434	5,634	46
76	Valle del Cauca	Palmira	5,794	5,614	47
73	Tolima	Ibagué	5,244	5,602	48
68	Santander	Girón	4,559	5,260	49
44	La Guajira	Barrancas	4,862	5,249	50
15	Boyacá	Tunja	4,831	5,109	51
08	Atlántico	Malambo	5,076	4,935	52
54	Norte de Santander	Chinácota	6,156	4,891	53
54	Norte de Santander	Ocaña	5,135	4,855	54
25	Cundinamarca	Mosquera	4,595	4,815	55
66	Risaralda	Dosquebradas	4,811	4,727	56
52	Nariño	Ipiales	3,954	4,626	57
17	Caldas	Manizales	4,612	4,569	58
50	Meta	Villavicencio	4,479	4,478	59
25	Cundinamarca	Madrid	4,189	4,336	60
**Subtotal (top 60 municipalities)**			**1,457,578**	**1,411,771**	
**Grand total (all municipalities)**			**1,771,237**	**1,729,537**	
**Population of top 60 as percentage of grand total**			**82%**	**82%**	
