# Supplementary material for: Impact of COVID-19 on healthcare utilization, cases, and deaths of citizens and displaced Venezuelans in Colombia: Complementary comprehensive and safety-net systems under Colombia’s constitutional commitment
Source: PLoS One. 2023 Mar 28;18(3):e0282786. doi: 10.1371/journal.pone.0282786 (PMC10047542; doi:10.1371/journal.pone.0282786)
Supplement: S2 File — (PDF) [file pone.0282786.s002.pdf]

SUPPORTING INFORMATION S2

Consistency over time of relative COVID-19 case rates

Supplement to

Impact of COVID-19 on healthcare utilization, cases, and deaths of citizens and displaced Venezuelans in Colombia: Complementary components of safety net and insurance systems under a constitutional commitment

In PLOS ONE 2023

by

Donald S. Shepard[1]

Adelaida Boada;[2]

Douglas Newball-Ramirez[2].

Anna G Sombrio[1]

Carlos William Rincon Perez[2]

Priya Agarwal-Harding[1]

Jamie S Jason[1]

Arturo Harker Roa[2]

Diana M. Bowser[1]

[1]The Heller School of Social Policy and Management, Brandeis University, Waltham, MA USA; [2] School of Government, Universidad de los Andes, Bogotá, Colombia

*Corresponding author: Donald S. Shepard, PhD, The Heller School for Social Policy and Management, MS035, Brandeis University, Waltham, Massachusetts 02454-9110, USA; email: shepard@brandeis.edu; Tel: +1-617-584-6664, ORCID: 0000-0003-2187-0593

February 28, 2023

To check consistency of relationships, the COVID-19 pandemic through November 2020 was subdivided into two almost equal time periods of 19 to 20 weeks each. Although Colombians had consistently higher COVID-19 rates than Venezuelans in the first time period, the rates were highly correlated (r=0.477) by municipality (Figure S2.1). Policies and practices around COVID-19 risk (e.g. masking and social distancing) as well as access to testing varied substantially among municipalities. When the rates were divided by quartile for Colombians, the log-ratio of highest quartile to the lowest quartile was 0.719 for Colombians and 0.573 for Venezuelans.

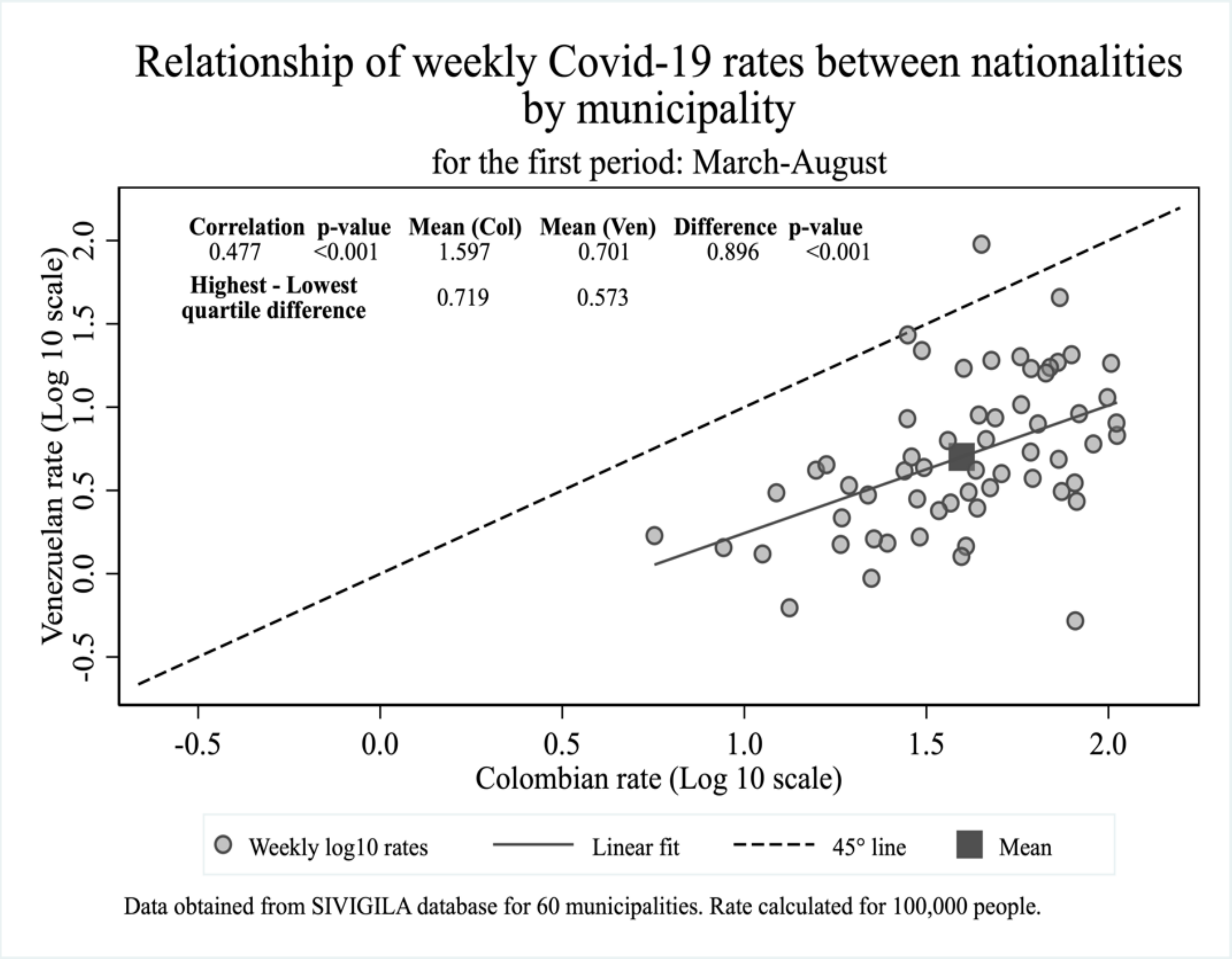

Figure S2.1. Analyses of COVID-19 case rates of Colombians and Venezuelans for the first period of 2020

Although Colombians had consistently higher COVID-19 rates than Venezuelans in the second time period, the rates were highly correlated (r=0.611) by municipality (Figure S2.2). Policies and practices around COVID-19 risk (e.g. masking and social distancing) as well as access to testing varied substantially among municipalities. When the rates were divided by quartile for Colombians, the log-ratio of highest quartile to the lowest quartile was 0.906 for Colombians and 1.021 for Venezuelans. The greater variation and higher correlation in the second period compared to the first is consistent with municipalities having more autonomy in setting COVID-19 policies after the national lockdown.

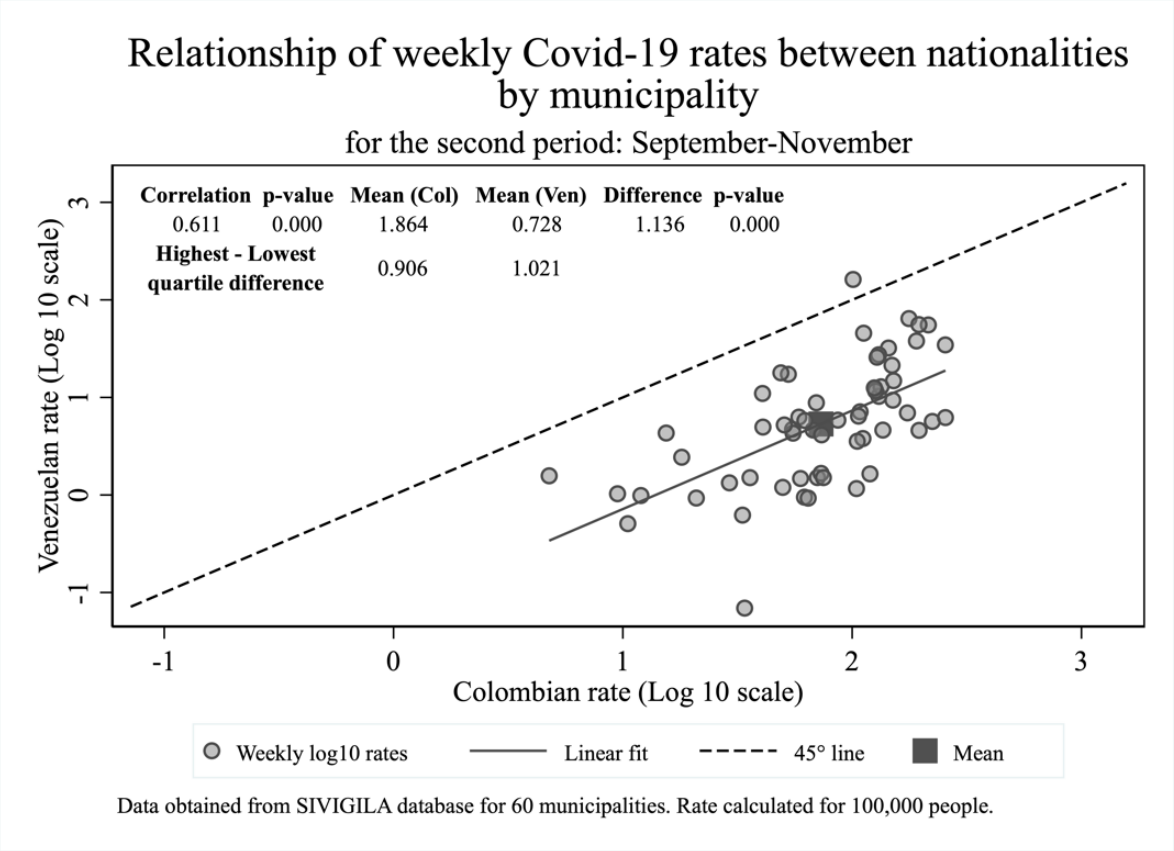

Figure S2.2. Analyses of COVID-19 case rates of Colombians and Venezuelans for the second period of 2020
