# Supplementary material for: Impact of COVID-19 on healthcare utilization, cases, and deaths of citizens and displaced Venezuelans in Colombia: Complementary comprehensive and safety-net systems under Colombia’s constitutional commitment
Source: PLoS One. 2023 Mar 28;18(3):e0282786. doi: 10.1371/journal.pone.0282786 (PMC10047542; doi:10.1371/journal.pone.0282786)
Supplement: S3 File — (PDF) [file pone.0282786.s003.pdf]

# SUPPORTING INFORMATION S3

**Impact of the pandemic on health services utilization by diagnosis**

**Supplement to**

**Impact of COVID-19 on healthcare utilization, cases, and deaths of citizens and displaced Venezuelans in Colombia: Complementary components of safety net and insurance systems under a constitutional commitment**

**In PLOS ONE 2023**

by

Donald S. Shepard[1]

Adelaida Boada;[2]

Douglas Newball-Ramirez[2].

Anna G Sombrio[1]

Carlos William Rincon Perez[2]

Priya Agarwal-Harding[1]

Jamie S Jason[1]

Arturo Harker Roa[2]

Diana M. Bowser[1]

[1]The Heller School of Social Policy and Management, Brandeis University, Waltham, MA USA; [2] School of Government, Universidad de los Andes, Bogotá, Colombia

*Corresponding author: Donald S. Shepard, PhD, The Heller School for Social Policy and Management, MS035, Brandeis University, Waltham, Massachusetts 02454-9110, USA; email: shepard@brandeis.edu; Tel: +1-617-584-6664, ORCID: 0000-0003-2187-0593

February 28, 2023

Figure S3.1 represents the percentage change of health services in each ICD-10 diagnosis chapter for the Venezuelan population in Colombia. The overall unweighted average change in utilization services for Venezuelans in these months from 2019 to 2020 was -19%, i.e., a decline of 19 percentage points or a ratio of 0.81 (i.e. 100% - 19%). This overall decline is broadly consistent with the ratio of 0.63 shown for Venezuelans at the top of Fig 4B of the main text. However, the numerical value differs because Figure S3.1 includes hospitalizations, which declined less than consultations, different scales (linear versus logarithmic), and weighted versus unweighted means. The diagnostic categories at the top of the graph show the ICD-10 diagnosis chapters with the largest declines for Venezuelans.

Conditions for which care could not be easily postponed (e.g., ICD-10 chapter 15, pregnancy, childbirth and puerperium) showed little change. Some conditions with little change (e.g., chapter 5, mental health) were likely exacerbated by the pandemic, but fear of infection likely suppressed an increase in service use. Finally, life threatening conditions (e.g., chapter 2 neoplasms, and chapter 9, circulatory conditions), saw relatively large increases as some Venezuelan migrants may have improved their ability to navigate the Colombian health care system. The absolute rates for Venezuelans are shown in Table S3.1.

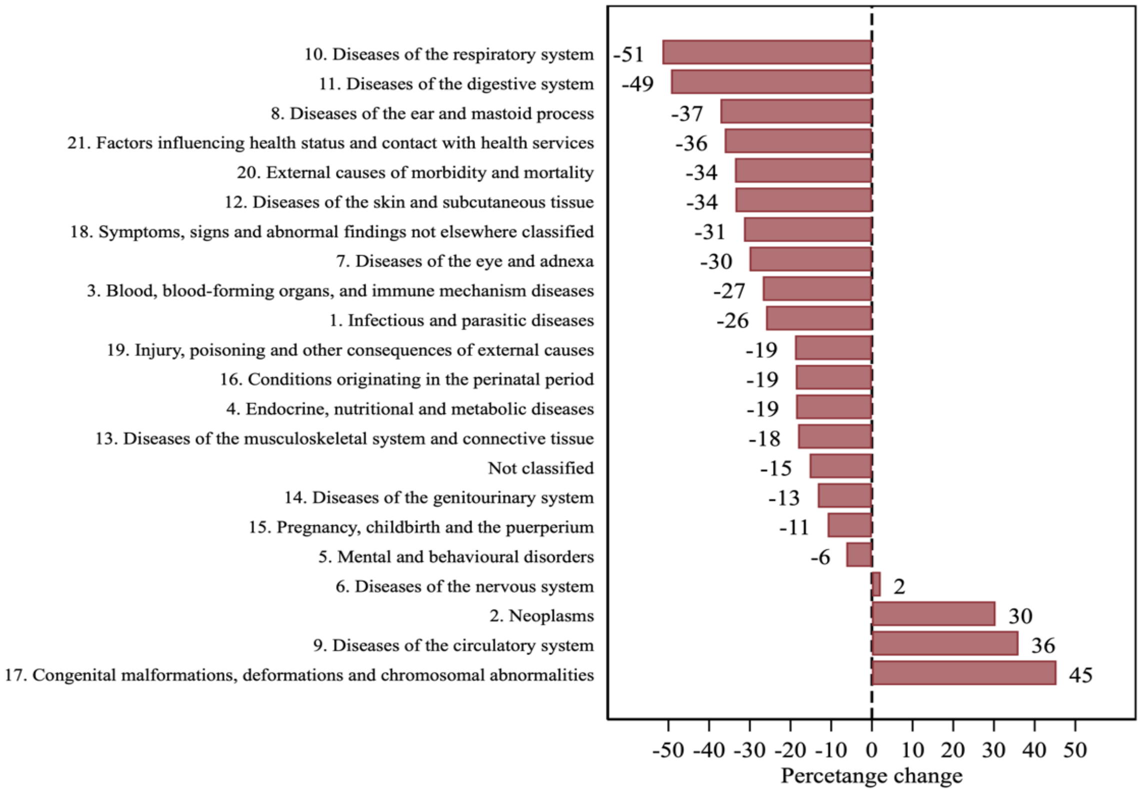


**Figure S3.1.** Change in utilization rate of health services from 2019 to 2020 of Venezuelans by ICD 10 diagnostic chapter

**Table S3.1.** Change in utilization rate of health services from 2019 to 2020 of Venezuelans by diagnostic categories

| ICD- 10 Chapter | Rate (2019) | Rate (2020) | % rate change (2020-2019) |
|---|---|---|---|
| 10. Diseases of the respiratory system | 2142 | 1039 | -51.5% |
| 11. Diseases of the digestive system | 2575 | 1304 | -49.4% |
| 8. Diseases of the ear and mastoid process | 161 | 101 | -37.2% |
| 21. Factors influencing health status and contact with health services | 10105 | 6450 | -36.2% |
| 20. External causes of morbidity and mortality | 156 | 104 | -33.6% |
| 12. Diseases of the skin and subcutaneous tissue | 601 | 399 | -33.5% |
| 18. Symptoms, signs and abnormal clinical and laboratory findings not elsewhere classified | 5152 | 3533 | -31.4% |
| 7. Diseases of the eye and adnexa | 145 | 102 | -30.1% |
| 3. Diseases of the blood and blood-forming organs and certain disorders involving the immune mechanism | 130 | 95 | -26.8% |
| 1. Certain infectious and parasitic diseases | 1426 | 1055 | -26.0% |
| 19. Injury, poisoning and certain other consequences of external causes | 2339 | 1897 | -18.9% |
| 16. Certain conditions originating in the perinatal period | 308 | 250 | -18.7% |
| 4. Endocrine, nutritional and metabolic diseases | 463 | 377 | -18.6% |
| 13. Diseases of the musculoskeletal system and connective tissue | 526 | 430 | -18.1% |
| 0. Not classified | 41067 | 34782 | -15.3% |
| 14. Diseases of the genitourinary system | 1558 | 1350 | -13.3% |
| 15. Pregnancy, childbirth and the puerperium | 5899 | 5257 | -10.9% |
| 5. Mental and behavioral disorders | 240 | 225 | -6.3% |
| 6. Diseases of the nervous system | 269 | 275 | 2.2% |
| 2. Neoplasms | 214 | 279 | 30.4% |
| 9. Diseases of the circulatory system | 377 | 513 | 36.0% |
| 17. Congenital malformations, deformations and chromosomal abnormalities | 54 | 79 | 45.4% |

Note: Rates of diagnosis from total utilization services from March through July of each year. Rates calculated per 100,000 population.

Using the same approach as for Venezuelans, Figure S3.2 represents the percentage change of health services for each ICD-10 diagnosis chapter for the Colombian population. The overall average change for Colombians was -35%, i.e., a decline of 35 percentage points, corresponding to a ratio of 0.65 (i.e., 100% - 35%). As with Venezuelans, this overall decline was broadly consistent with the ratio of 0.58 in the main text. The numerical values differs slightly between the main text 8 because Figure S3.2 includes hospitalizations, which declined less than consultations, different scales (linear versus logarithmic), and weighted versus unweighted means. The diagnosis chapters at the top of the graph, which show large declines for Colombians, also reflect declines in the true incidence of the underlying condition and/or avoidance of medical treatment of that condition. For example, chapter 11 (diseases of the digestive system) may have benefited from improved hygiene (hand washing and cleaning surfaces), as with Venezuelans. Similarly, chapter 10 (diseases of the respiratory system) likely declined because the social distancing to protect against COVID-19 also protected Colombians against other respiratory illnesses. Chapter 7 (diseases of the eye) reflects conditions for which care could often be safely postponed. The 24% decline in mental health conditions (chapter 5) was below the previously mentioned overall decline (35%), perhaps signaling challenges in accessing services for non-life-threatening problems during the pandemic.

Pregnancy services (chapter 15), which could not be safely postponed, declined relatively little (-20%). Life threatening conditions e.g., neoplasms (chapter 2) and circulatory system diseases (chapter 9) showed relatively little decline, perhaps because Colombians' appreciation of the risks from these diseases outweighed their fear of contracting COVID-19 in the course of medical care. The absolute rates for Colombians are shown in Table S3.2.

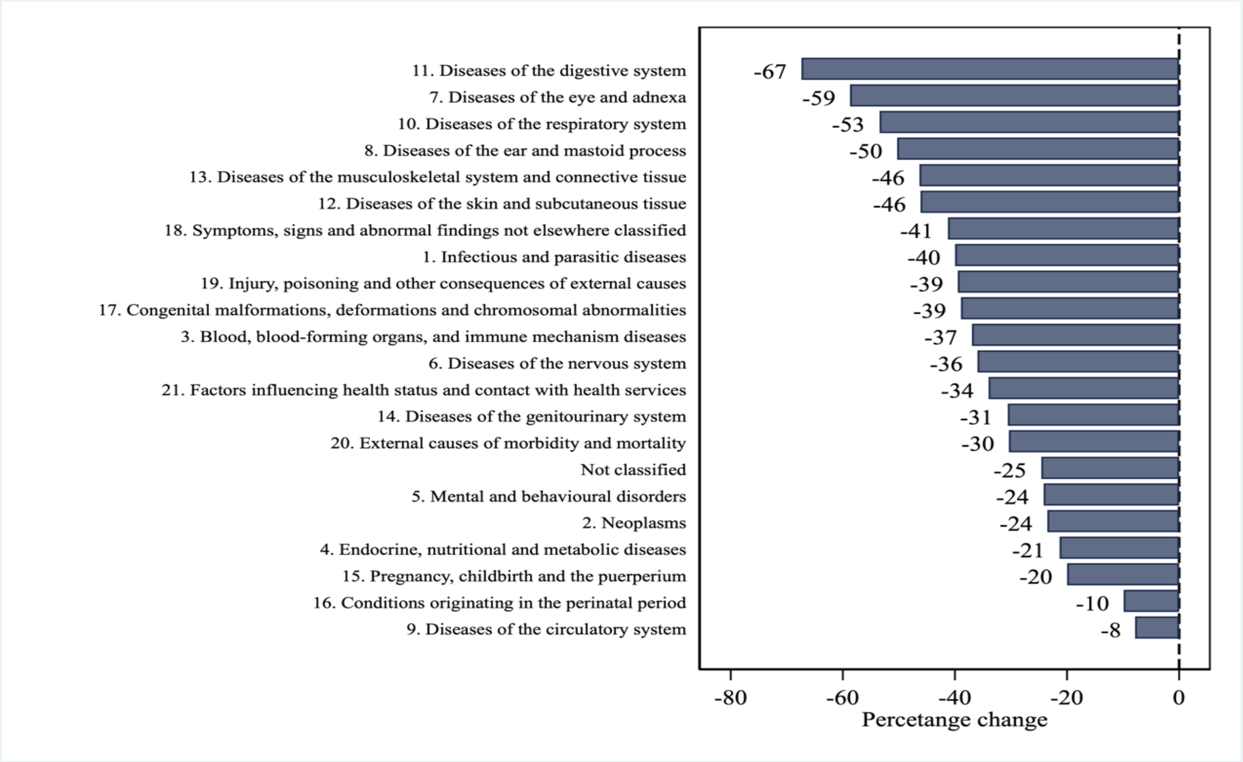


**Figure S3.2.** Change in utilization rate of health services from 2019 to 2020 of Colombians by ICD-10 diagnostic chapter

**Table S3.2.** Change in utilization rate of health services from 2019 to 2020 of Colombians by diagnostic categories

| ICD-10 Chapter | Rate (2019) | Rate (2020) | % rate change (2020-2019) |
|---|---|---|---|
| 11. Diseases of the digestive system | 38468 | 12547 | -67.4% |
| 7. Diseases of the eye and adnexa | 8055 | 3325 | -58.7% |
| 10. Diseases of the respiratory system | 15305 | 7127 | -53.4% |
| 8. Diseases of the ear and mastoid process | 3246 | 1613 | -50.3% |
| 13. Diseases of the musculoskeletal system and connective tissue | 19009 | 10202 | -46.3% |
| 12. Diseases of the skin and subcutaneous tissue | 5969 | 3216 | -46.1% |
| 18. Symptoms, signs and abnormal clinical and laboratory findings not elsewhere classified | 43016 | 25273 | -41.2% |
| 1. Certain infectious and parasitic diseases | 9816 | 5892 | -40.0% |
| 19. Injury, poisoning and certain other consequences of external causes | 10904 | 6599 | -39.5% |
| 17. Congenital malformations, deformations and chromosomal abnormalities | 1177 | 719 | -38.9% |
| 3. Diseases of the blood and blood-forming organs and certain disorders involving the immune mechanism | 1606 | 1013 | -37.0% |
| 6. Diseases of the nervous system | 6655 | 4261 | -36.0% |
| 21. Factors influencing health status and contact with health services | 87519 | 57765 | -34.0% |
| 14. Diseases of the genitourinary system | 16070 | 11157 | -30.6% |
| 20. External causes of morbidity and mortality | 2099 | 1462 | -30.4% |
| 0. Not classified | 78110 | 58902 | -24.6% |
| 5. Mental and behavioral disorders | 7144 | 5418 | -24.2% |
| 2. Neoplasms | 6211 | 4751 | -23.5% |
| 4. Endocrine, nutritional and metabolic diseases | 16627 | 13082 | -21.3% |
| 15. Pregnancy, childbirth and the puerperium | 3538 | 2831 | -20.0% |
| 16. Certain conditions originating in the perinatal period | 698 | 629 | -9.9% |
| 9. Diseases of the circulatory system | 27607 | 25439 | -7.9% |

Note: Rates of diagnosis from total utilization services from March through July of each year. Rates calculated per 100,000 population.

## S5. Effect of insurance regime

Figure S5.1 shows the proportion (at the municipality level) of healthcare use that is provided through the contributory regime, versus its COVID-19 rate, by municipality. In general, the higher that portion, the higher the population and the greater the level of institutional, social and economic development of that municipality (see Supplementary Information S6). There are positive correlations between COVID-19 rates and the share of contributory regime participation in total healthcare use for both Venezuelans and Colombians across municipalities. These results suggest that in municipalities with larger percentages of healthcare services through the contributory regime, there was more COVID-19 testing and treatment, and therefore higher rates of reported COVID-19 cases

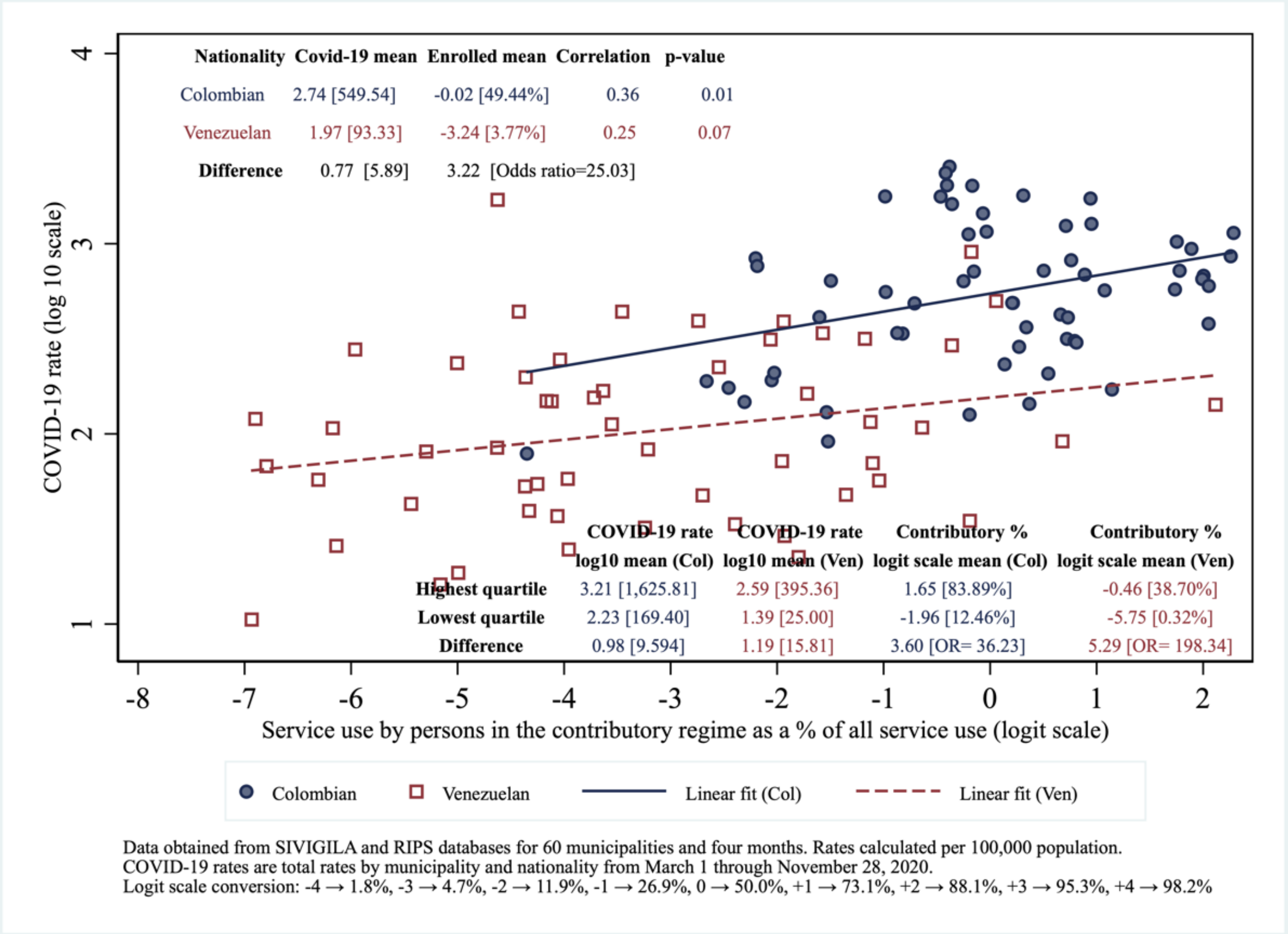


**Figure S5.1.** Health plan (EPS) contributory regime share vs COVID-19 rate of Colombians and Venezuelans

Figure S5.2 shows relative use of health services through the contributory regime versus hospitalization rates. The overall rate of hospitalizations is remarkably similar between the two nationalities. While Colombians had 1.55 times the hospitalization rate compared to Venezuelans, this difference is small compared to the 10-fold differences seen in COVID-19 rates. These patterns also apply separately to the first and second periods of pandemic in 2020 (see Supplementary Information S2). These findings suggest that the Colombian health care system has done a good job of implementing the constitutional requirement to ensure access to urgent care of all residents in the country, regardless of nationality or regime status. This result means that both populations have access to urgent health care regardless of whether they are affiliated

to the contributory regime, the subsidized regime, or if they are not enrolled at all. While the interquartile variation in contributory share is substantial for both Venezuelans (factor of 198) and Colombians (factor of 36), the insignificant correlations (p-values of 0.86 and 0.17) suggest that the contributory share was not an important determinant of hospitalization rates. Under the alternative specification of overall enrollment (or affiliation), the relationship to hospitalization rates became negative (see Figure S5.2). This result suggests that service use in the subsidized population was closer to that of the unenrolled than to the contributory members".

Regression analyses below (Supplementary Information S6) found that the population size in a municipality does not affect its rate of hospitalizations. To illustrate this finding, we have labeled the points corresponding to Colombia's three largest municipalities in Figure S5.2 As expected, their share of contributory enrollment was near or above the grand mean for all municipalities. However, their hospitalization rates were not appreciably different from overall mean rate for the same nationality. Thus, while hospitalization rates vary across Colombia, the variation is related to factors other than population size.

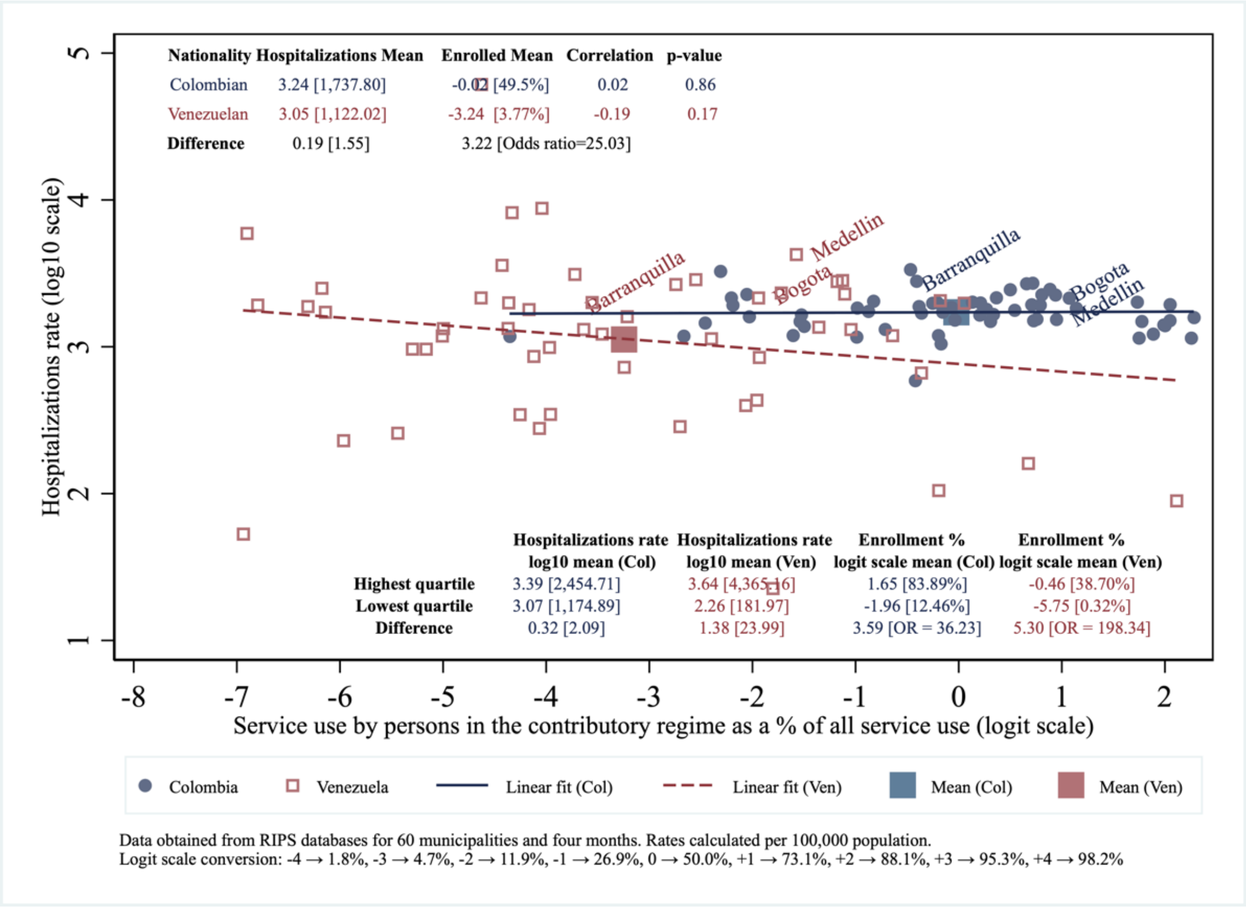


**Figure S5.2.** Health plan (EPS) contributory regime enrollment vs hospitalization rates of Colombians and Venezuelans

Figure S5.3 examines how access to the healthcare system affects rates of consultations. This figure shows that there is a positive and highly significant correlation between consultation rates and average contributory regime participation (at the municipality level) for Colombians, across municipalities. This

correlation is also positive for Venezuelans, but it is not statistically significant. These patterns also generally apply separately to the first and second segments of the pandemic in 2020 (see Supplementary Information S2). These results support the conclusion that both populations have better access to health care in relatively more developed contexts – in terms of the quality of institutions, rule of law, economic activity and labor market formality. As with the analyses of hospitalizations, after controlling for the relative enrollment to the contributory regime, Colombians have a dramatically higher rate of consultations (by a factor of 7.08) than Venezuelans. The findings for Colombia's three largest municipalities (labeled in Figure S5.3) are consistent with those for hospitalizations. As expected, their share of contributory enrollment was near or above the grand mean for all municipalities. However, their consultation rates were not appreciably different from the grand means.

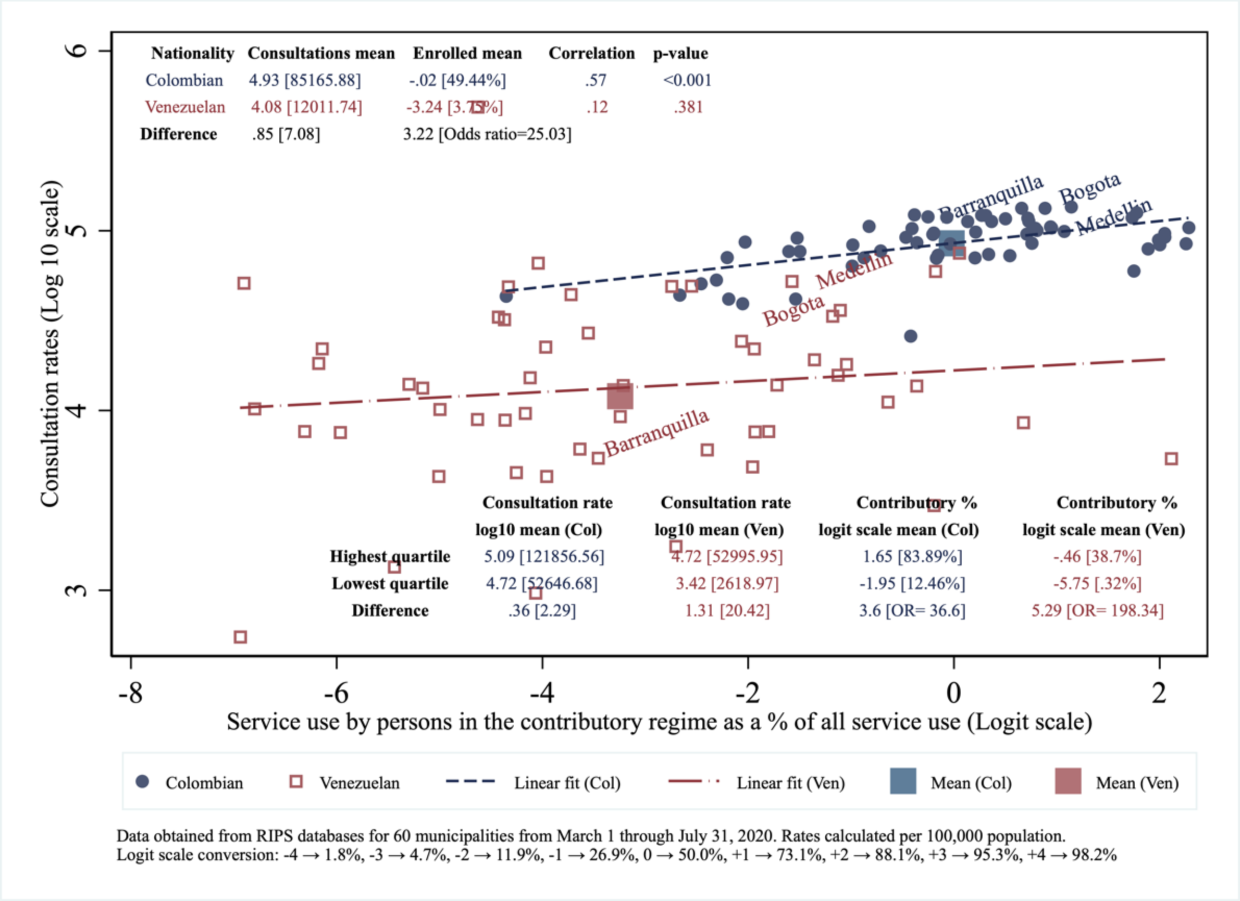


**Figure S5.3.** Health plan (EPS) contributory regime enrollment vs consultation rates of Colombians and Venezuelans

## S6. Relationship of city size to health services utilization

Table S6.1 shows the effect of the population size of a municipality against several measures of utilization and insurance coverage by municipality.

**Table S6.1.** Ordinary least squares (OLS) regression estimation of health services use against city size

| | Regression (dependent variable and nationality examined) | | | | | |
|---|---|---|---|---|---|---|
| Independent Variables | Colombians' hospitalization rates (log10 scale) | Venezuelans' hospitalization rates (log10 scale) | Colombians' consultation rates (log10 scale) | Venezuelans' consultation rates (log10 scale) | Colombians' contributory affiliation % (logit scale) | Venezuelans' contributory affiliation % (logit scale) |
| Total population (log10 scale) | 0.0193 | 0.114* | 0.0642*** | 0.190*** | 0.537*** | 0.683*** |
| | (0.0154) | (0.0668) | (0.0162) | (0.0590) | (0.156) | (0.255) |
| Constant | 2.996*** | 1.639* | 4.137*** | 1.731** | -6.662*** | -11.77*** |
| | (0.192) | (0.828) | (0.201) | (0.732) | (1.933) | (3.187) |
| Observations | 60 | 60 | 60 | 60 | 60 | 54 |
| R-squared | 0.026 | 0.048 | 0.213 | 0.152 | 0.170 | 0.122 |

Notes: *** p<0.01, ** p<0.05, * p<0.1. Standard errors in parentheses. Data obtained from RIPS databases for 60 municipalities and four months. Rates calculated per 100,000 population.
