# Supplementary material for: Impact of COVID-19 on healthcare utilization, cases, and deaths of citizens and displaced Venezuelans in Colombia: Complementary comprehensive and safety-net systems under Colombia’s constitutional commitment
Source: PLoS One. 2023 Mar 28;18(3):e0282786. doi: 10.1371/journal.pone.0282786 (PMC10047542; doi:10.1371/journal.pone.0282786)
Supplement: S4 File — (PDF) [file pone.0282786.s004.pdf]

SUPPORTING INFORMATION S4

Effect of insurance regime on rates of COVID-19 cases, hospitalization, and consultations

Supplement to:

Impact of COVID-19 on healthcare utilization, cases, and deaths of citizens and displaced Venezuelans in Colombia: Complementary components of safety net and insurance systems under a constitutional commitment

In PLOS ONE 2023

by

Donald S. Shepard[1]

Adelaida Boada;[2]

Douglas Newball-Ramirez[2].

Anna G Sombrio[1]

Carlos William Rincon Perez[2]

Priya Agarwal-Harding[1]

Jamie S Jason[1]

Arturo Harker Roa[2]

Diana M. Bowser[1]

[1]The Heller School of Social Policy and Management, Brandeis University, Waltham, MA USA; [2] School of Government, Universidad de los Andes, Bogotá, Colombia

*Corresponding author: Donald S. Shepard, PhD, The Heller School for Social Policy and Management, MS035, Brandeis University, Waltham, Massachusetts 02454-9110, USA; email: shepard@brandeis.edu; Tel: +1-617-584-6664, ORCID: 0000-0003-2187-0593

Effect of insurance regime on COVID-19 case rates

Figure S5.1 shows the proportion (at the municipality level) of healthcare use that is provided through the contributory regime, versus its COVID-19 rate, by municipality. In general, the higher that portion, the higher the population and the greater the level of institutional, social and economic development of that municipality (see Supplementary Information S6). There are positive correlations between COVID-19 rates and the share of contributory regime participation in total healthcare use for both Venezuelans and Colombians across municipalities. These results suggest that in municipalities with larger percentages of healthcare services through the contributory regime, there was more COVID-19 testing and treatment, and therefore higher rates of reported COVID-19 cases

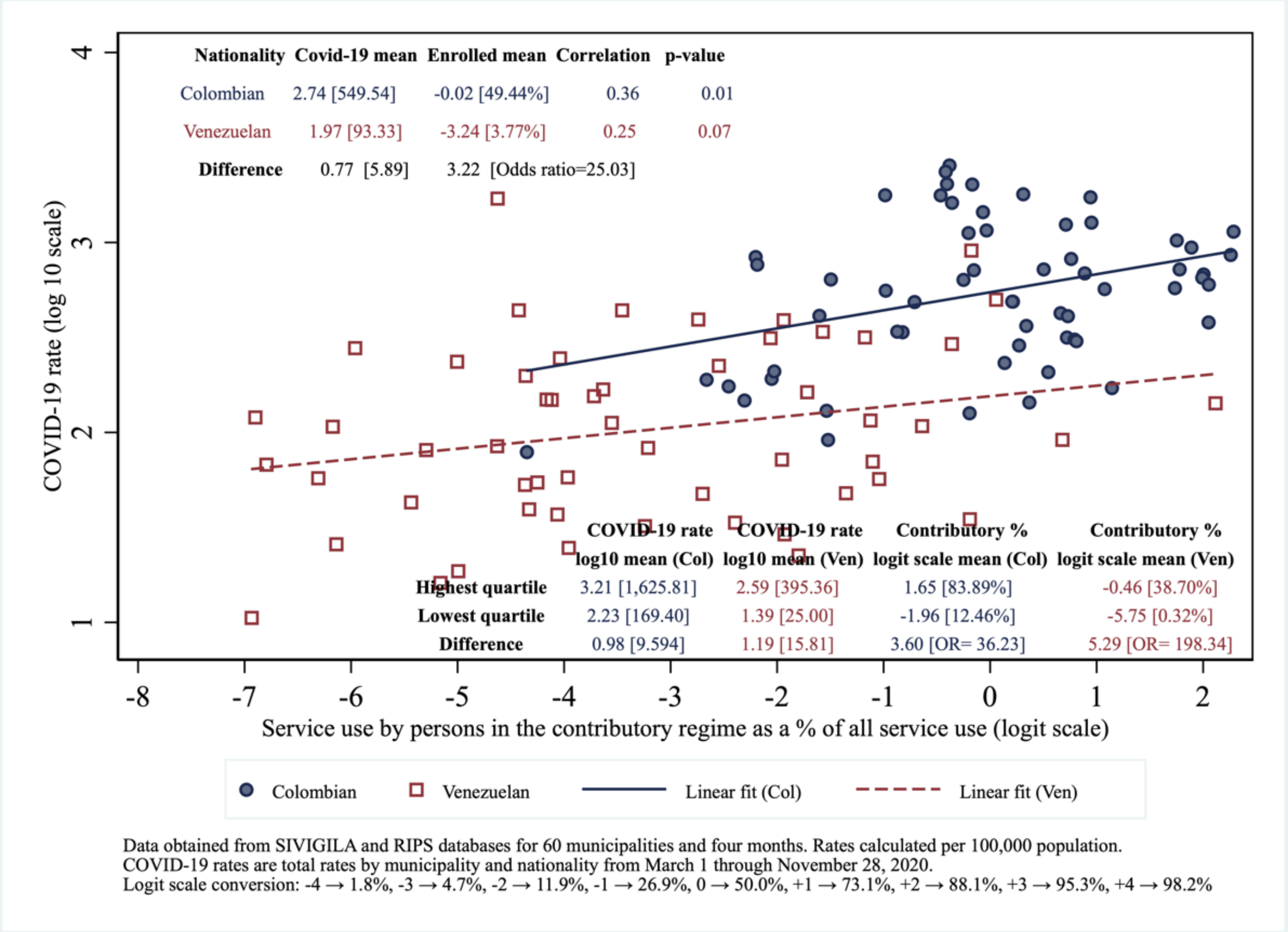

Figure S5.1. Health plan (EPS) contributory regime share vs COVID-19 rate of Colombians and Venezuelans

Effect of insurance regime on hospitalization rates

Figure S5.2 shows relative use of health services through the contributory regime versus hospitalization rates. The overall rate of hospitalizations is remarkably similar between the two nationalities. While Colombians had 1.55 times the hospitalization rate compared to Venezuelans, this difference is small compared to the 10-fold differences seen in COVID-19 rates. These patterns also apply separately to the first and second periods of pandemic in 2020 (see Supplementary Information S2). These findings suggest that the Colombian health care system has done a good job of implementing the constitutional requirement to ensure access to urgent care of all residents in the country, regardless of nationality or regime status. This result means that both populations have access to urgent health care regardless of whether they are affiliated to the contributory regime, the subsidized regime, or if they are not enrolled at all. While the interquartile variation in contributory share is substantial for both Venezuelans (factor of 198) and Colombians (factor of 36), the insignificant correlations (p-values of 0.86 and 0.17) suggest that the contributory share was not an important determinant of hospitalization rates. Under the alternative specification of overall enrollment (or affiliation), the relationship to hospitalization rates became negative (see Figure S5.2). This result suggests that service use in the subsidized population was closer to that of the unenrolled than to the contributory members".

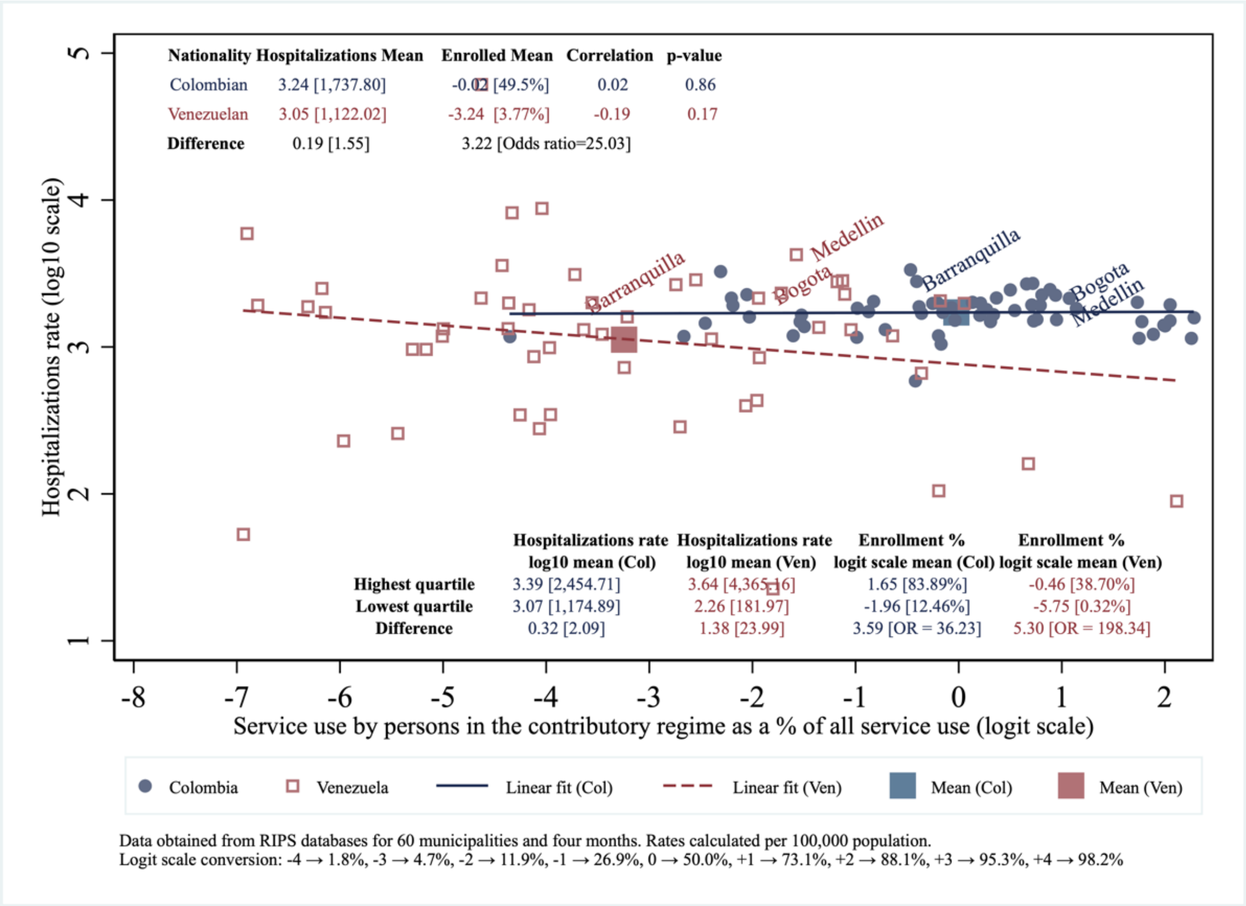

Figure S5.2. Health plan (EPS) contributory regime enrollment vs hospitalization rates of Colombians and Venezuelans

Regression analyses below (Supporting Information S5) found that the population size in a municipality does not affect its rate of hospitalizations. To illustrate this finding, we have labeled the points corresponding to Colombia's three largest municipalities in Figure S5.2 As expected, their share of contributory enrollment was near or above the grand mean for all municipalities. However, their hospitalization rates were not appreciably different from overall mean rate for the same nationality. Thus, while hospitalization rates vary across Colombia, the variation is related to factors other than population size.

Effect of insurance regime on consultation rates

Figure S5.3 examines how access to the healthcare system affects rates of consultations. This figure shows that there is a positive and highly significant correlation between consultation rates and average contributory regime participation (at the municipality level) for Colombians, across municipalities. This correlation is also positive for Venezuelans, but it is not statistically significant. These patterns also generally apply separately to the first and second segments of the pandemic in 2020 (see Supplementary Information S2). These results support the conclusion that both populations have better access to health care in relatively more developed contexts – in terms of the quality of institutions, rule of law, economic activity and labor market formality. As with the analyses of hospitalizations, after controlling for the relative enrollment to the contributory regime, Colombians have a dramatically higher rate of consultations (by a factor of 7.08) than Venezuelans. The findings for Colombia's three largest municipalities (labeled in Figure S5.3) are consistent with those for hospitalizations. As expected, their share of contributory enrollment was near or above the grand mean for all municipalities. However, their consultation rates were not appreciably different from the grand means.

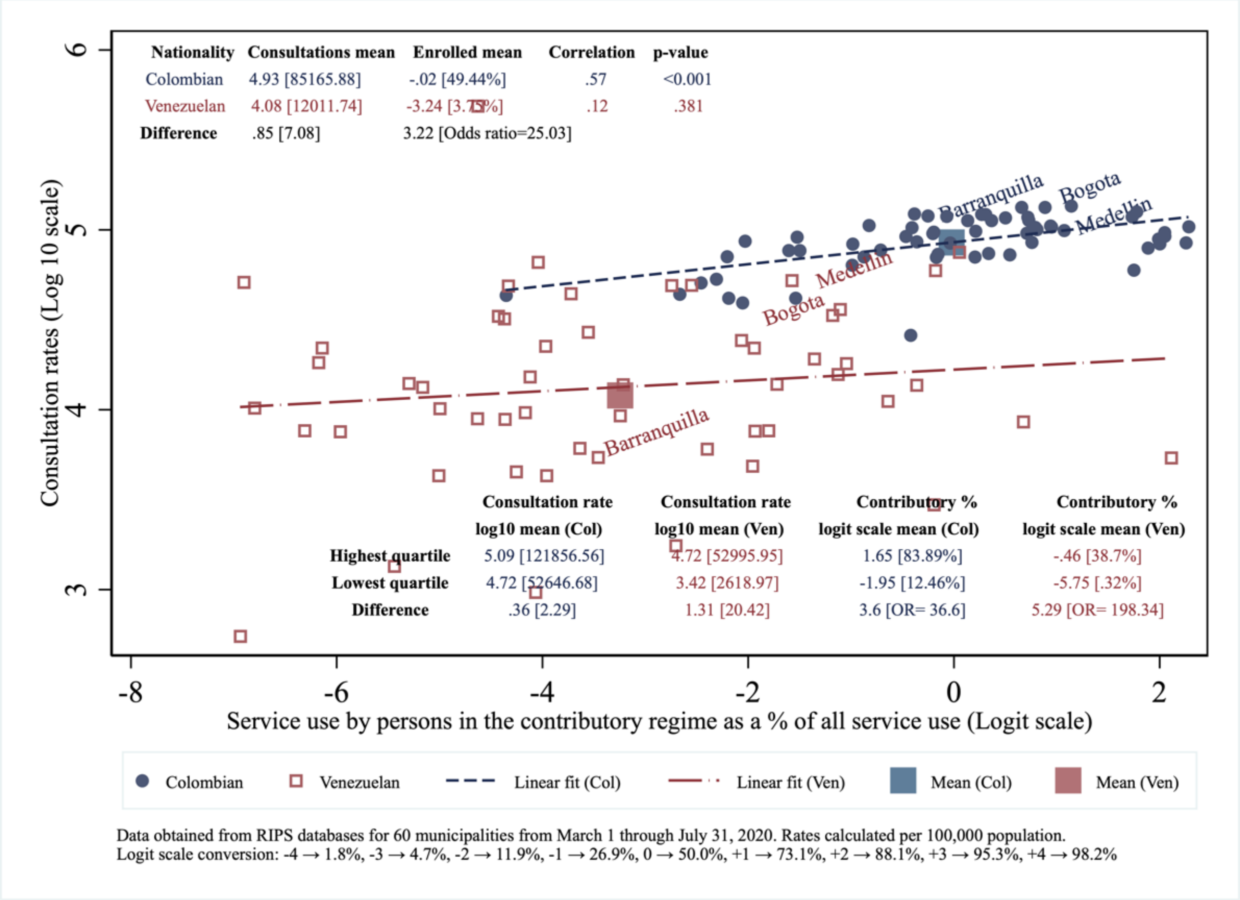

Figure S5.3. Health plan (EPS) contributory regime enrollment vs consultation rates of Colombians and Venezuelans
