# Supplementary material for: Impact of COVID-19 on healthcare utilization, cases, and deaths of citizens and displaced Venezuelans in Colombia: Complementary comprehensive and safety-net systems under Colombia’s constitutional commitment
Source: PLoS One. 2023 Mar 28;18(3):e0282786. doi: 10.1371/journal.pone.0282786 (PMC10047542; doi:10.1371/journal.pone.0282786)
Supplement: S5 File — (PDF) [file pone.0282786.s005.pdf]

SUPPORTING INFORMATION S5

Relationship of city size to health services utilization

Supplement to:

Impact of COVID-19 on healthcare utilization, cases, and deaths of citizens and displaced Venezuelans in Colombia: Complementary components of safety net and insurance systems under a constitutional commitment

In PLOS ONE 2023

by

Donald S. Shepard[1]

Adelaida Boada;[2]

Douglas Newball-Ramirez[2].

Anna G Sombrio[1]

Carlos William Rincon Perez[2]

Priya Agarwal-Harding[1]

Jamie S Jason[1]

Arturo Harker Roa[2]

Diana M. Bowser[1]

[1]The Heller School of Social Policy and Management, Brandeis University, Waltham, MA USA; [2] School of Government, Universidad de los Andes, Bogotá, Colombia

*Corresponding author: Donald S. Shepard, PhD, The Heller School for Social Policy and Management, MS035, Brandeis University, Waltham, Massachusetts 02454-9110, USA; email: shepard@brandeis.edu; Tel: +1-617-584-6664, ORCID: 0000-0003-2187-0593

February 28, 2023

Table S5.1 shows the effect of the population size of a municipality against several measures of utilization and insurance coverage by municipality.

Table S5.1. Ordinary least squares (OLS) regression estimation of health services use against city size

	Regression (dependent variable and nationality examined)					
Independent Variables	Colombians' hospitalization rates (log10 scale)	Venezuelans' hospitalization rates (log10 scale)	Colombians' consultation rates (log10 scale)	Venezuelans' consultation rates (log10 scale)	Colombians' contributory affiliation % (logit scale)	Venezuelans' contributory affiliation % (logit scale)
Total population (log10 scale)	0.0193	0.114*	0.0642***	0.190***	0.537***	0.683***
	(0.0154)	(0.0668)	(0.0162)	(0.0590)	(0.156)	(0.255)
Constant	2.996***	1.639*	4.137***	1.731**	-6.662***	-11.77***
	(0.192)	(0.828)	(0.201)	(0.732)	(1.933)	(3.187)
Observations	60	60	60	60	60	54
R-squared	0.026	0.048	0.213	0.152	0.170	0.122

Notes: *** p<0.01, ** p<0.05, * p<0.1. Standard errors in parentheses. Data obtained from RIPS databases for 60 municipalities and four months. Rates calculated per 100,000 population.
