# Supplementary material for: Impact of COVID-19 on healthcare utilization, cases, and deaths of citizens and displaced Venezuelans in Colombia: Complementary comprehensive and safety-net systems under Colombia’s constitutional commitment
Source: PLoS One. 2023 Mar 28;18(3):e0282786. doi: 10.1371/journal.pone.0282786 (PMC10047542; doi:10.1371/journal.pone.0282786)
Supplement: Attachment — Submitted filename: Comments_to_authors_PONE-D-22-2128_v1.pdf [file pone.0282786.s007.pdf]

Manuscript reference: PONE-D-22-21282

Manuscript title: "Impact of COVID-19 on healthcare utilization, cases, and deaths of citizens and displaced Venezuelans in Colombia: Complementary components of safety net and insurance systems under a constitutional commitment"

Overview

This study assesses the healthcare utilization outcomes (hospitalization, consultation) and death rates during the pandemic times period (2020) and pre-pandemic period (2019) among Colombian citizens and Venezuelan people who emigrated to Colombian due to the Venezuelan political and socio-economic conditions. In addition, the author sought to identify potential disparities within and between the studied population concerning the studied outcomes.
I think the study has the potential to add important evidence on the healthcare-related condition and inequalities/ equities of Venezuelan emigrants in Colombia. However, the paper needs a better structure, and important detailed information in all sections (introduction, methods, discussions) to improve its quality and potential impact. Also, some statements are not backed by the current study or existing data, which requires careful addressing from the authors. The text requires careful proofing/editing revisions as well. Colombian adjectives need to be used (as you are referring to the Colombian context)instead of Columbian ones (which do not relate to the Colombian Country)

Thank you for the opportunity to review the paper, and here below, I provide my comments to the authors, that hopefully could contribute to improving their manuscript.

Abstract

1. The abstract should have structured headings: Background or Objective, Method, Results, and Conclusion.
2. "We compared utilization of comprehensive (primarily consultations) and safety net (primarily hospitalization) services and deaths between citizens and Venezuelans in Colombia." I think the exact nationality of the citizens needs to be stated: For example, between Colombian citizens and Venezuelans in Colombia.
3. "Using national databases",The specific type of database should be stated.
4. What exact months are included within the mid-2020 statement? I think this is important to better understand if the study was carried out during the first wave of the COVID-19 pandemic or later.
5. 60 municipalities: Colombian municipalities? Also, briefly state within brackets the definition of the municipality.
6. Statistical or analysis methods used should be stated.
7. I think the results, should be structured in a much clear way.

Ethics Statement

8. The country or jurisdiction of the Institutional Research Board that approved the study needs to be stated.

Introduction

9. Lines 49-51. Global Fund countries. Please, briefly explain what means that expression or classification, especially as you then refer to the “Fund’s target diseases.” Also, relate to the latter; please, state to which three exact diseases you are referring.
10. Lines 51-54. Please specify if you are referring to healthcare services, social support services, or both in such a statement. Also, identify which are those other problems. Are those “other problems”, non-covid-19 health concerns people experience, such as traditional chronic diseases, and/or surgery, primary care, etc.?
11. Line 55, please provide some examples of the healthcare challenges that recipient countries experience.
12. Similarly, in lines 59/60, please complete such a statement, especially what services are those demanding from local citizens and displaced people.
13. Lines 65/66. Please provide the main reasons why the pandemic has inadvertently reduced access to care with the associated reference(s).
14. Line 67, I think the authors are referring to the Colombian Context; therefore, “Columbian” is a misspelling word” of the Colombian adjective.
15. Lines 68/69 briefly provide the main reasons for the Venezuelan people's displacement. It helps to understand and contextualize the problem and compare them with those displaced around the globe, and even with the Colombian internally displaced people.
16. Line 71, did they only search for work opportunities? Or safe and more served settings as well?
17. Line 75. The word “consultation”, what is it referring to? Physician consultations, any contact with the healthcare systems, etc.? Also, provide the timeline for when a such referenced study was conducted and the specific bibliographic reference.
18. Line 81. Please, provide the date of the beginning of the Venezuelan migratory crisis.
19. Line 83, what are such specific benefits?
20. Lines 83 to 85, despite that statement is important to highlight, IT seems incomplete in the sense or connection with the Colombian addressing approach of Venezuelan displaced people living in its territory. Perhaps, the authors meant that that policy is based on a sort of gratitude or support for the Venezuelan people due to the support they provided to Colombian people who started to be displaced due to the internal Colombian conflict and drug trafficking-related violence in 1990. Therefore, the Colombian government is now providing the same support to the Venezuelan displaced due to socio-economic or political reasons.
21. Line 88, please describe the type of health system coverage that Colombia has, which will help to understand the issues analyzed in this paper. From my knowledge is a hybrid insurance-based public-private health care system rather than a totally public personal health service as stated in line 91.
22. Line 91, see my previous comment.

23. Paragraphs 91 to 102. It is also important to state that beyond the main three mechanisms to access healthcare in Colombia, many people in Colombia have added private health insurance or paid out of their pocket for private healthcare services.

Methods

24. Lines 114 and 116. Insurance coverage is repeated twice in the same sentence; therefore, amend or differentiate such expression. Also, in line 114, “quality of services,” please specify what type of services you are referring to. Are those healthcare services, social services, etc.?
25. Among the barrier to services, you only stated two; lockdown restrictions and facility staffing shortage. What about the lack of medical/treatment supplies and appropriate and equipped medical facilities?
26. Line 142, consultation: are there physician consultations? Or any consultation with a health professional? Inpatient or outpatient consultation? Please specify it.
27. Line 114, the list of the 60 municipalities, should be provided at least as supplementary information.
28. Regarding the health care utilization rates(lines 146-156), what kind of rate do you include: incident rates or count-based (repeated) rates? Also, were they calculated from the overall March 1 through July 31, 2020? Or in a monthly based interval (Monthly rates) over such an overall study period?
29. Why were emergency department visits not considered? Emergency department visits are considered an important indicator of healthcare needs, utilization and accessibility, especially among underserved population groups.
30. COVID-19 cases and deaths and overall deaths. The authors stated that they have no reason to suspect any widespread or systematic errors in the death data. What about misreporting issues, both underreported and overreported COVID-19 death data?
31. Please provide the death data period used. For example, was the same data period for healthcare utilization outcomes?
32. Analytical approach, rates per 100,000 population, are those for hospitalization, consultation incident or repeated events rates?
33. Is there any reason the chosen death-related period differed from the healthcare utilization period?
34. What statistical models or methods did you use to compare the rates of healthcare utilization and deaths across the two compared populations and pre-and post-pandemic periods?

Ethical exemption

35. As stated in a previous comment, the country or jurisdiction of the Institutional Research Board (REB) that approved the study needs to be stated. Also, why any Colombian-based REB was involved?

Results

36. Lines 223-224. Please specify the two periods that you segmented and compared, as it is not clear from the provided information.

37. Line 232. What are those similar factors? Shared area-based factors? Individual factors?
38. Lines 243 and 245. Where did those results remain after being adjusted by age? As it is likely that there are other factors beyond age that may explain such differences, such as COVID-19 exposure-related factors, health status, sex and gender.
39. Lines 285 and 286. Also, they may be likely due to the prioritization of COVID-19-related healthcare over traditional services for non-COVID-19 health issues as a strategy to reduce the healthcare system collapse. This may also be related to the healthcare staffing shortage. Again, however, the interpretations can be moved to the discussion rather than have them in the result.
40. 294, (by 42% (1.00-0.58)) is the within bracket figures a confidence interval or interquartile range?
41. Line 313 to 314. Again, the interpretation of findings should be left for discussion.
42. Is there any correlation between consultation and hospitalization for each studied population group? For example, where are the rates of consultation correlated to the hospitalization rates in the Colombian and Venezuelan populations?
43. Finally, is there any reason biological sex or gender, as well as other socio-economic factors such as education level, and health status, were not considered in the analyses?

Discussion

44. Lines 336 to 340, despite age, can be one of the explanations. It is likely that biological sex and gender also influence the findings. The authors stated genocide as a potential cause of Venezuelan migration, but I would be very cautious of using that word without evidence. Yet, it is important to recognize that Venezuelans have suffered different and systematic human rights violations. However, I don't think the study findings and type of study can be used to make the genocide assertion.
45. Also, regarding the healthy immigrant effect, it is important to highlight that such an effect could reduce with time when migrants assimilate local environmental or behavioural factors. Resilience can be another explanatory factor. https://link.springer.com/article/10.1007/s10903-021-01313-5
46. Lines 358, there is likely a huge misreporting effect among Venezuelans due to different factors, such as slow access to technologies (PC, Smartphone) to access healthcare services virtually, which were common during the pandemic. Also, it is likely that due to fear of being discriminated against and stigmatized as a potential virus spreader and as a high utilizer of healthcare services and social services, and the high criminalization they have experienced (https://www.crisisgroup.org/latin-america-caribbean/andes/colombia-venezuela/hard-times-safe-haven-protecting-venezuelan ; https://www.unhcr.org/en-ie/61f40a914.pdf, https://globalizationandhealth.biomedcentral.com/articles/10.1186/s12992-020-00655-3), Venezuelans may have used fewer healthcare services and reported fewer COVID-19 symptoms. Also, in the Colombian context, people still have socio-economic and limited access to COVID-19 testing; therefore, underreporting is expected.
47. Line 366, the assertion "while Colombians, knowing that they had good access to comprehensive care" are not backed for any reference. As a Colombian, I rather believe that many Colombians, especially those in the middle or lower social class, have a lot of

complex and interrelated socio-economic unmet needs; therefore, they cannot afford not to go out and look for survival resources, including working even without appropriate personal protection equipment, exposing them to a higher risk of contracting COVID-19.
https://jech.bmj.com/content/76/2/116
https://www.ncbi.nlm.nih.gov/pmc/articles/PMC7934198/
https://www.ncbi.nlm.nih.gov/pmc/articles/PMC8940255/

48. Lines 377-379. I think the study's data do not support this statement. Despite the Colombian government's efforts to strengthen the health services resources during the pandemic pick, people still experienced limited access to health services for many non-covid-19 problems. https://www.frontiersin.org/articles/10.3389/fpubh.2022.896318/full The Colombian government also had serious limitations in implementing key health initiatives, such as early vaccination, which could reduce the severity of COVID-19 cases and associated death. https://pubmed.ncbi.nlm.nih.gov/35340774/
49. Among the recommendations, strategies to tackle discrimination and stigma against Venezuelans in the Colombian context should be considered at the individual, population and structural levels, including healthcare settings, to decrease potential health and social inequalities in Venezuelans in Colombia.
50. "With international support, Colombia's well-designed national health system could address many of these needs. However, this statement is so general. What do you mean by Colombia's well-designed national health system?
51. A limitations section needs to be included.
