# Supplementary material for: Impact of COVID-19 on healthcare utilization, cases, and deaths of citizens and displaced Venezuelans in Colombia: Complementary comprehensive and safety-net systems under Colombia’s constitutional commitment
Source: PLoS One. 2023 Mar 28;18(3):e0282786. doi: 10.1371/journal.pone.0282786 (PMC10047542; doi:10.1371/journal.pone.0282786)
Supplement: Attachment — Submitted filename: plos-one-review4.docx [file pone.0282786.s006.docx]

**Responses to requests from PlosOne editors**

A. **Ethical approval.** The authors have now added a section at the end of Methods entitled “Ethical exemption” at line 210 providing full details of the official exemption from human studies consent procedures.
B. **Information about a previous brief.** The authors have added a sentence to the end of the manuscript at lines 461-463 as follows: “These recommendations build on a brief from an earlier version of this study that was posted online but not peer reviewed.[32].” That citation contains the full title and link to the online brief.
C. **Rebuttal to review of previous manuscript**. As requested, the authors have provided the following rebuttal to the decision letter from Plos One regarding our previous related manuscript. The previous manuscript was entitled “Differential impact of the COVID‐19 pandemic in Colombia on utilization of medical services between Venezuelan migrants and citizens.” The decision letter (PONE‐D‐21‐30924) was signed by Renuka Sane, Academic Editor, and sent on Dec. 10., 2021. The authors thank the academic editor and reviewers for the constructive comments. For each item, the reviewers’ comments (labeled “reviewers”) have been quoted. The authors’ response to each comment follows and begins with the label “authors.”
1. Reviewers: Is the manuscript technically sound, and do the data support the conclusions?

The manuscript must describe a technically sound piece of scientific research with data that supports the conclusions. Experiments must have been conducted rigorously, with appropriate controls, replication, and sample sizes. The conclusions must be drawn appropriately based on the data presented.

Reviewer #1: Partly

Reviewer #2: No

Authors: The authors believe their revisions now provide the necessary rigor. The paper has been retitled and substantially restructured and rewritten. First, the main theme is now systematically comparing two care systems – a safety net system versus a comprehensive system. The authors now compare services related to each of these subsystems, showing that Venezuelans generally had only the safety net system, while Colombians enjoyed the comprehensive system.

Second, the authors have obtained and carefully analyzed mortality data, as described further in the comments below. These added data allow distinguishing artifacts in health services utilization between real differences in age-adjusted outcomes between Venezuelan refugees and age matched standards.

2.	Reviewers: Has the statistical analysis been performed appropriately and rigorously?

Reviewer #1: Yes

Reviewer #2: No

Authors: In analyses of mortality and utilization, the authors have added significance tests to examine whether differences between Colombians and Venezuelans are statistically significant.

3. Reviewers: Have the authors made all data underlying the findings in their manuscript fully available?

The PLOS Data policy requires authors to make all data underlying the findings described in their manuscript fully available without restriction, with rare exception (please refer to the Data Availability Statement in the manuscript PDF file). The data should be provided as part of the manuscript or its supporting information, or deposited to a public repository. For example, in addition to summary statistics, the data points behind means, medians and variance measures should be available. If there are restrictions on publicly sharing data—e.g. participant privacy or use of data from a third party—those must be specified.

Reviewer #1: No

Reviewer #2: Yes

Authors: The authors have added links to the public web sites from which data on service utilization, mortality and population can be obtained.

4. Reviewers: Is the manuscript presented in an intelligible fashion and written in standard English?
5. PLOS ONE does not copyedit accepted manuscripts, so the language in submitted articles must be clear, correct, and unambiguous. Any typographical or grammatical errors should be corrected at revision, so please note any specific errors here.

Reviewer #1: No

Reviewer #2: No

Authors: The manuscript has been extensively edited by the authors as well as by a professional editor for clarity. To ensure adherence to the journal’s guidelines, the figures have been removed from the text and added as separate graphical files.

5 Reviewers: Comments to the Author

5.1 Reviewer #1: Thank you for submitting your article on one of the important aspects of the Colombian health system. You may please have a look at the following comments:

Overall, the manuscript requires better formatting and language correction. Your introduction section might improve by adding adequate referencing. For example, on page 7, lines 125‐127 would read much better if you provided evidence for it.

Authors: We thank the reviewer for this suggestion. The material referenced (background on data sources) has been moved to the methods section. If now begins at line 136. Citations have been added for each data source.

5.2 Reviewers: Framing of the research question could be done better. The write up is too long. It reads like a descriptive report. It lacks the focus of a research article. The discussion section can improve by situating the findings to previous literature. It lacks the rigour of an article.

Authors: As noted above, the paper has been restructured and retitled to highlight the contrast between the safety net and comprehensive systems.

5.3 Reviewers: The conclusion section may be more focused and based on the findings only. Finally, the reference section of the manuscript is mainly based on reports.

Authors: To improve the linkage of conclusions with the rest of the report, we no longer have a separate “conclusions” section. Instead, we have a subsection of the discussion entitled “suggested policy changes.” To focus the discussion more clearly, we have inserted section titles and inserted citations to peer-reviewed publications, such as the healthy immigrant effect (see line 331) and global literature on the relationship between aging and COVID-19 risks (see line 305). In a number of cases the most current information was available only from reports and online sources. We have, however, added peer reviewed publications wherever possible. One example is reference 8, written by some of the same researchers as the present manuscript. Another example is reference 23 about the “healthy immigrant effect” to explain why Venezuelan migrants had relatively low age-specific mortality.

5.4 Reviewer #2: Comments on “Differential impact of the COVID‐19 pandemic in Colombia on utilization of medical services between Venezuelan migrants and citizens”

The authors use administrative data from Colombia to examine changes in healthcare utilization across Colombian natives and Venezuelan immigrants in Colombia pre and post the Covid‐19 pandemic. Specifically, the goal of the article is to understand whether access to healthcare services has been more limited for Venezuelans in Colombia during the pandemic. Thus, they compare rates of covid19 hospitalizations, deaths and non‐covid19 hospitalizations and consultations across the two population groups and over time.

They find that relative to 2019, in 2020, hospitalization rates per 100k population fell for both Colombians and Venezuelans but fell more sharply for Colombians. At the same time, they find that Colombians have higher Covid19 cases per 100k and deaths per 100k. In terms of non‐covid19 care, Colombians face larger declines in consultations post pandemic. Overall, the authors conclude that this evidence together indicates that Venezuelans had access to essential healthcare services during the pandemic.

Authors: We agree with this synopsis of the original submission.

5.5	Reviewer 2’s major comments

1. The article is poorly written. It’s extremely verbose yet unclear. Several theories are implicitly tested or commented on at the same time. It’s a little chaotic and does not read well.
2. The evidence from the descriptive statistics (I consider all figures as descriptive statistics) does not lead to the conclusions in the manuscript. Particularly, here are some sentences I have copied and pasted that were perplexing to read. These sentences are beyond the scope of the data (I elaborate more on what the data does and does not tell us below)

Examples of Large claims made:

a. “The comparable 2020 rates of hospitalization between the two nationalities indicates that the Colombian health system has fulfilled its constitutional mandate to provide emergency care for the neediest members of both nationalities” .

In contrast to the quote above and in the same article, the authors also argue that testing was limited for Venezuelans. They say “Colombians’ higher 41 rates of documented COVID‐19 cases and deaths likely reflects their greater access to 42 ambulatory care and COVID‐19 testing”. So how did the constitutional mandate then provide emergency care for the neediest members? Again, this is not backed by the data or the findings and is born out of thin air.

b. “Colombia’s decision in 2021 to allow most Venezuelans a ten‐year residence permit, should help lower disparities in ambulatory service use by promoting migratory status regularization.”

Is this an opinion? how does the article comment on the ten‐year residence permit program directly if it does not examine this particular program?

Authors: As noted above, the manuscript has been restructured, extensively revised, and professionally edited. In rewriting the paper, the offending sentences above have been removed. The authors have endeavored to provide the facts and bases for their conclusions.

5.6 Reviewer 2’s suggestion. Instead What should be examined:

a. If the question is whether the Colombian healthcare system managed to remain accessible to Venezuelans during the pandemic, then one could examine all excess deaths and non‐covid19 excess deaths among Venezuelans vs Colombians over time. The assumption here is that the non‐covid19 excess deaths are a result of missed or deferred care. If it is examined then it’s a direct measure to answer whether those who needed care were serviced. They only examine covid‐ 19 deaths. They also argue that differences in covid19‐death rates reflect rates of testing and documentation across the two groups. So they are not that useful.
b. Instead of examining non‐covid19 excess deaths, the authors compare rates of hospitalizations and consultations pre- and post pandemic for each group. I comment on this approach below:
   i. Prior to the pandemic the consultation rates for Colombians are higher. Meaning they use more care but also presumably a different margin of care. Eg: they may use more elastic or less clinically significant care too. After the pandemic, we notice that Venezuelans reduce care and so do Colombians – but the Colombians by more. The authors then conclude that this is evidence that Venezuelans were still able to access care (because their decrease was lower). See figure 5 for consultations.
   ii. The issue however is a 1% reduction in care for Colombians is not equivalent to a 1% decrease in care for Venezuelans. If the Venezuelan population was ex‐ante consuming more inelastic and clinically undeferrable care, then a 1% decrease is much more detrimental (either in the short run or in the long run). So comparing the magnitudes of the decreases in my opinion is a pointless exercise.

For example, consider a scenario where Colombians had wasteful pre‐pandemic care that they get rid of or shed during the pandemic.

iii. The same argument can be made about hospitalizations. A 1% decrease in hospitalizations for Venezuelans is not measuring the same medical inputs as a 1% decrease in hospitalizations for Colombians.
iv. The best approach in my opinion is to examine excess deaths across the two groups for non‐covid19 type illnesses. That would be the direct approach to understanding whether the Venezuelan group faced barriers to access and a widening disparity. That is not done here.

Authors: The authors thank the reviewer for these insights and the suggestion to examine non-COVID deaths. The revised manuscript implemented them insofar as the data allowed. The reviewer’s hypothesis that the Colombians were consuming more elastic or less clinically significant care is consistent with their higher rates of utilization. Unfortunately, available data do not allow testing that hypothesis. The only available data are numbers of services by the chapter (first digit) of the disease classification. These cannot distinguish the first visit for a life threatening condition versus, say, an unnecessary fifth visit for a minor problem.

On the other hand, the authors were able to operationalize the reviewer’s suggestion to examine death rates. Instead of non-COVID deaths, we examined crude and age-adjusted death rates of Colombians and Venezuelans. While we had overall deaths by age group, nationality and municipality, COVID deaths by nationality and municipality were available only in aggregate, not age-specific. These aggregate data showed that non-COVID deaths were 99.6% of Colombians’ crude death rate (530.1 per 100,000) and 98.1% of Venezuelans’ crude death rate (36.5 per 100,000). Thus, in aggregate, rates of total deaths and non-COVID-19 deaths were nearly identical. To examine non-COVID deaths specifically by municipality would have required forgoing age standardization. Given how much younger the Venezuelans were compared to the Colombians, inability to standardize deaths by age category would have been a major limitation.

The authors analysis of age-standardized mortality data showed a positive correlation between years, as expected, suggesting that persistent municipality factors affected both years and the data appear usable. The changes in age-standardized mortality from 2019 to 2020 found that lower and declining rates for Venezuelans compared to higher and rising rates for Colombians. These findings are consistent with a good safety net system for Venezuelans, although other explanations must also be considered (see p 19, lines 322ff).

6. PLOS authors have the option to publish the peer review history of their article (what does this mean?). If published, this will include your full peer review and any attached files. If you choose “no”, your identity will remain anonymous but your review may still be made public.
7. Reviewers: Do you want your identity to be public for this peer review? For information about this choice, including consent withdrawal, please see our Privacy Policy.

Reviewer #1: No

Reviewer #2: No
