# Supplementary material for: Impact of COVID-19 on healthcare utilization, cases, and deaths of citizens and displaced Venezuelans in Colombia: Complementary comprehensive and safety-net systems under Colombia’s constitutional commitment
Source: PLoS One. 2023 Mar 28;18(3):e0282786. doi: 10.1371/journal.pone.0282786 (PMC10047542; doi:10.1371/journal.pone.0282786)
Supplement: Attachment — Submitted filename: Comments_to_authors_PONE-D-2128_v137 DS.docx [file pone.0282786.s008.docx]

**Manuscript reference**: PONE-D-22-21282

# Manuscript title: “Impact of COVID-19 on healthcare utilization, cases, and deaths of citizens and displaced Venezuelans in Colombia: Complementary comprehensive and safety-net systems under Colombia’s constitution”

Response: Thank you for the opportunity to revise and resubmit our manuscript. For clarity, we have introduced a decimal numbering for all comments, using E for the editor’s comments, Q for Reviewer 1’s questions and R for Reviewer 2’s comments. We have responded to each comment, noting the approximate line number in the revised (clean) version where the corresponding revision begins.

**Editor**

E1. The manuscript tackles an important topic that deserves to be highlighted and shared. However, it looks more like a report than a research article. The authors are given a chance to improve the writing and the structure of the manuscript. The paper needs a serious revision before it is accepted. The authors are invited to address very carefully all comments provided by the two reviewers.

E1 Response. We have made extensive comments based on the constructive comments from the editor and reviewers.

E2a. I also strongly recommend that authors ‐during their revision‐ follow the STROBE checklist for reporting of observational studies. Please remove subheadings from the introduction and let the research question be clearly stated at the end of the introduction: what is the aim of the study?

E2a Response. In the limitations section, we noted that our study observations are aggregate data for each of 2 nationalities within each of 60 municipalities. Thus, the specific frameworks for observational studies at the individual level do not apply. We have, however, discussed our steps to minimize bias and the direction of possible remaining biases. Please see lines 526 to 536.

E2b. Some information about the context in the introduction can be moved to the materials and methods under "study setting and participants". Please follow the STROBE checklist when choosing the subheadings in "Materials and Methods".

E2b Response. We moved material from the introduction and created a subheading under Materials and Methods entitled “Setting,” at line 121. Please see comment E2a for further discussion about STROBE.

E2c. On the other hand, the language needs careful and thorough revision; it is surprising that "Colombia" is often misspelled and written "Columbia".

E2c Response. We apologize for the misspelling and have corrected that error. We have also carefully reviewed and revised the manuscript.

E2d. Finally, it is necessary to add a "limitations" paragraph to the discussion.

E2d Response. We have added a limitation section starting on line 510.

E3. Thank you for submitting the above manuscript to PLOS ONE. During our internal evaluation of the manuscript, we found significant text overlap between your submission and previous work in the Introduction, Methods, Results and Discussion sections of your manuscript. We would like to make you aware that copying extracts from previous publications, especially outside the methods section, word‐for‐word is unacceptable. In addition, the reproduction of text from published reports has implications for the copyright that may apply to the publications. Please revise the manuscript to rephrase the duplicated text, cite your sources, and provide details as to how the current manuscript advances on previous work. Please note that further consideration is dependent on the submission of a manuscript that addresses these concerns about the overlap in text with published work. We will carefully review your manuscript upon resubmission and further consideration of the manuscript is dependent on the text overlap being addressed in full. Please ensure that your revision is thorough as failure to address the concerns to our satisfaction may result in your submission not being considered further.

E3 Response. Thank you for this honest comment. First, I want to confirm that my co-authors and I understand that text cannot be copied verbatim from previous publications. We are committed to upholding this standard. To our knowledge, the revised manuscript does not copy any substantial text from previous publications. To ensure transparency, we have cited all previous publications and online documents. Our two previous publications appeared in *Health Systems and Reform* (on the telephone survey, reference 10) and *BMC Public Health* (on mobility, reference 15). The online working paper was issued by UNDP (reference 16). We also wrote a Knowledge Brief about health service utilization which is posted on the web site of Columbia University, the university which coordinated the consortium of studies of which this is a component (reference 12). However, the Knowledge Brief is not a publication, nor was it peer reviewed or copyrighted. It is not in a book, report, or journal. In many ways, it is similar to a blog posted on the authors’ university web site. As a final step to avoid potential repetition, we completely rewrote the introduction section and ensured sufficient changes elsewhere. The markings in the tracked version document the extensive revisions.

E4.	Thank you for stating in your Funding Statement:

“This study was primarily funded by the project "Strengthening the humanitarian response to COVID‐19 in Colombia" through a grant to Brandeis University from ELRHA (https://www.elrha.org/), with DMB as PI, which supported all authors. Additional funding was provided by the World Bank (www.worldbank.org) through award WORLD 8006362 to Columbia University, Sub‐award 2(PG006778‐01) to Brandeis University (DSS, Brandeis PI) for the project "The big questions on forced migration in health." It supported DSS, PA‐H, JSS, AHR, and DMB. The funders had no role in study design, data collection and analysis, decision to publish or preparation of the manuscript.” Please provide an amended statement that declares *all* the funding or sources of support (whether external or internal to your organization) received during this study, as detailed online in our guide for authors at http://journals.plos.org/plosone/s/submit‐now. Please also include the statement “There was no additional external funding received for this study.” in your updated Funding Statement.

E4 Response. The revised funding statement has been entered into the cover letter and follows:

“This study was primarily funded by the project "Strengthening the humanitarian response to COVID‐19 in Colombia" through a grant to Brandeis University from ELRHA (https://www.elrha.org/), with DMB as PI, which supported all authors. The remaining funding was provided by the World Bank (www.worldbank.org) through award WORLD 8006362 to Columbia University, Sub‐award 2(PG006778‐01) to Brandeis University (DSS, Brandeis PI) for the project "The big questions on forced migration in health." It supported DSS, PA‐H, JSS, AHR, and DMB. The funders had no role in study design, data collection and analysis, decision to publish or preparation of the manuscript. There was no additional external funding received for this study.”

E5.	We note that you have included the phrase “data not shown” in your manuscript. Unfortunately, this does not meet our data sharing requirements. PLOS does not permit references to inaccessible data. We require that authors provide all relevant data within the paper, Supporting Information files, or in an acceptable, public repository. Please add a citation to support this phrase or upload the data that corresponds with these findings to a stable repository (such as Figshare or Dryad) and provide and URLs, DOIs, or accession numbers that may be used to access these data. Or, if the data are not a core part of the research being presented in your study, we ask that you remove the phrase that refers to these data.

E5 Response. We have created a document of Supplementary Information which we would request would be provided online if our paper is published. One section of this, S2, provides the breakdowns by periods within 2020. We have replaced the phrase “Data not shown” by the reference to the Supplementary Information S2. We also added other sections of Supplementary Information for a total of 6 sections to further support PLOS One’s principle of data access and transparency.

E6 (sent 14 Feb 2023). We note that parts of your manuscript were published as knowledge brief. To increase transparency, please clearly indicate at the end of the Introduction section that the content of this manuscript has also been included in a knowledge brief and provide the relevant reference.

E6 Response. We added a sentence to the end of the introduction and the citation (reference 12) on lines 82-83.

**Reviewer #1**

Q1. The authors made a significant effort to attend to points raised by the reviewers in the manuscript's previous version. The work done is methodologically correct and brings some interesting findings. However, I think that it still lacks scientific density, and contributes little to a more comprehensive discussion about healthcare access by migrants and refugees worldwide, a challenging and relevant issue.

The references are still mostly reports instead of international scientific articles focused on the central thema, which would probably allow for putting the Colombian experience in a more compheensive perspective, able to contribute to building knowledge about the thema. Based in the literature, it would be important to confront the Colombian experience with the Venezuelans with other countries' experiences with migrants and refugees.

Which healthcare systems' models are more responsive to the needs of migrants and refugees? What are the implications for host countries, considering different levels of resources' availability?

Q1 Response. Thanks for this important question. We added a paragraph to the discussion comparing Colombia to four other sites in the consortium. Please see the paragraph beginning on line 476.

Q2.	With regard to the writing, some problems still remain.

Q2 Response. The manuscript has been carefully checked and revised. We hope we have addressed all the problems.

**Reviewer 2**

**Overview**

**This study assesses the healthcare utilization outcomes (hospitalization, consultation) and death** rates during the pandemic times period (2020) and pre-pandemic period ( 2019 ) among Colombian citizens and Venezuelan people who emigrated to Colombian due to the Venezuelan political and socio-economic conditions. In addition, the author sought to identify potential disparities within and between the studied population concerning the studied outcomes. I think the study has the potential to add important evidence on the healthcare-related condition and inequalities/ equities of Venezuelan emigrants in Colombia. However, the paper needs a better structure, and important detailed information in all sections (introduction, methods, dcussions) to improve its quality and potential impact. Also, some statements are not backed by the current study or existing data, which requires careful addressing from the authors. The text requires careful proofing/editing revisions as well. Colombian adjectives need to be used (as you are referring to the Colombian context )instead of Columbian ones ( which do not relate to the Colombian Country)

Thank you for the opportunity to review the paper, and here below, I provide my comments to the authors, that hopefully could contribute to improving their manuscript.

R0. The very detailed and constructive comments from this reviewer (Cilia Mejia Lancheros—she agreed to release her name) were very helpful and much appreciated.

# Abstract

1. The abstract should have structured headings: Background or Objective, Method, Results, and Conclusion.

R01 Response. This was done. The Abstract begins on line 20

2. “We compared utilization of comprehensive (primarily consultations) and safety net (primarily hospitalization) services and deaths between citizens and Venezuelans in Colombia.” I think the exact nationality of the citizens needs to be stated: For example, between Colombian citizens and Venezuelans in Colombia.

R02 Response. This clarification was added – see the abstract starting on line 27.

3. “Using national databases”,The specific type of database should be stated.

R03 Response. We added “health services, population, disease surveillance, and deaths’ to the Methods section in the abstract – please see line 29.

4. What exact months are included within the mid-2020 statement? I think this is important to better understand if the study was carried out during the first wave of the COVID-19 pandemic or later.

R04 Response. We added “March through November 2020” – please see line 30.

5. 60 municipalities: Colombian municipalities? Also, briefly state within brackets the definition of the municipality.

R05 Response. We added “local governments” in parentheses – see line 28.

6. Statistical or analysis methods used should be stated.

R06 Response. We added “We employed ratios, log transformations, correlations, and regressions using national databases for population, health services, disease surveillance, and deaths.” – please see line 28.

7. I think the results, should be structured in a much clear way.

R07 Response. Results were rewritten.

8. Ethics Statement. The country or jurisdiction of the Institutional Research Board that approved the study needs to be stated.

R08 Response. We added “based in the United States.” Please see line 265.

# Introduction

9. Lines 49-51. Global Fund countries. Please, briefly explain what means that expression or classification, especially as you then refer to the “Fund’s target diseases.” Also, relate to the latter; please, state to which three exact diseases you are referring.

R09 Response. We added: AIDS, malaria and tuberculosis – see line 53.

10. Lines 51-54. Please specify if you are referring to healthcare services, social support services, or both in such a statement. Also, identify which are those other problems. Are those “other problems”, non-covid-19 health concerns people experience, such as traditional chronic diseases, and/or surgery, primary care, etc.?

R10 Response. We added for clarification “non-COVID concerns.” See line 60

11. Line 55, please provide some examples of the healthcare challenges that recipient countries experience.

R11 Response. We added examples and the citation to the final report from a related project -lines 61-63 and added reference 3 - the World Bank Consortium.

12. Similarly, in lines 59/60, please complete such a statement, especially what services are those demanding from local citizens and displaced people.

R12 Response. We added “for healthcare, social services, shelter and security.” Please see line 68.

13. Lines 65/66. Please provide the main reasons why the pandemic has inadvertently reduced access to care with the associated reference(s).

R13 Response. As the preceding sentence gave specifics and references, we deleted the sentence with “inadvertently reduced.”

14. Line 67, I think the authors are referring to the Colombian Context; therefore, “Columbian” is a misspelling word” of the Colombian adjective.

R14 Response. Thanks for catching that. With research partners at Columbia University, we inadvertently confounded the words.

15. Lines 68/69 briefly provide the main reasons for the Venezuelan people's displacement. It helps to understand and contextualize the problem and compare them with those displaced around the globe, and even with the Colombian internally displaced people.

R15 Response. In the methods subsection “Setting,” we added “escape Venezuela’s economic collapse,” provided additional context, and a citation – see lines 124-127.

16. Line 71, did they only search for work opportunities? Or safe and more served settings as well?

R16 Response. In the methods subsection “Setting,” we added “searching for relative safety and work opportunities”– see lines 126.”

17. Line 75. The word “consultation”, what is it referring to? Physician consultations, any contact with the healthcare systems, etc.? Also, provide the timeline for when a such referenced study was conducted and the specific bibliographic reference

R17 Response. We defined “consultation.” To clarify our timeline, we added the months and year in each figure in the main text and tables in the Supplementary Information in its title or legend. We also expanded our citations to previous publications and online resources, termed “family of studies.” Please see line 128.

18. Line 81. Please, provide the date of the beginning of the Venezuelan migratory crisis.

R18 Response. The sentences were rewritten indicating July 2015 as the start – see line 124.

19. Line 83, what are such specific benefits?

R19 Response. We replaced this phrase by the more specific benefit “apply to enroll in an EPS….” See line 507

20. Lines 83 to 85, despite that statement is important to highlight, IT seems incomplete in the sense or connection with the Colombian addressing approach of Venezuelan displaced people living in its territory. Perhaps, the authors meant that that policy is based on a sort of gratitude or support for the Venezuelan people due to the support they provided to Colombian people who started to be displaced due to the internal Colombian conflict and drug trafficking-related violence in 1990. Therefore, the Colombian government is now providing the same support to the Venezuelan displaced due to socio-economic or political reasons.

R20 Response. We added a sentence about gratitude for past assistance. – Please see text beginning with line 136

21. Line 88, please describe the type of health system coverage that Colombia has, which will help to understand the issues analyzed in this paper. From my knowledge is a hybrid insurance-based public-private health care system rather than a totally public personal health service as stated in line 91.

R21 Response. We added the term IPS and discussed its funding. Although employers and workers are funders of the national insurance system, we would still classify the method of funding as public. Our reason is that contributions are collected through a payroll tax rather than premium payments. We noted that private insurance and funding also exist. IPS is referred to beginning at line 175.

22. Line 91, see my previous comment.

R22 Response. See R21

23. Paragraphs 91 to 102. It is also important to state that beyond the main three mechanisms to access healthcare in Colombia, many people in Colombia have added private health insurance or paid out of their pocket for private healthcare services.

R23 Response. We added a sentence about privately funded healthcare. Please see line 185.

# Methods

24. Lines 114 and 116. Insurance coverage is repeated twice in the same sentence; therefore, amend or differentiate such expression. Also, in line 114, “quality of services,” please specify what type of services you are referring to. Are those healthcare services, social services, etc.?

R24 Response. We clarified by saying “healthcare services.” Please see line 99.

25. Among the barrier to services, you only stated two; lockdown restrictions and facility staffing shortage. What about the lack of medical/treatment supplies and appropriate and equipped medical facilities?

R25 Response. Thanks the suggestions. We added “shortages of medical supplies and qualified workers, and disruptions in service delivery.” Please see lines 58-60

26. Line 142, consultation: are there physician consultations? Or any consultation with a health professional? Inpatient or outpatient consultation? Please specify it.

R26 Response. Where this term was first used in the text, we added the following explanation: Consultations are formal contacts with a medical professional, such as a physician, for diagnosis and possible treatment in a non-emergency ambulatory setting. This begins on line 85 See also R17.

27. Line 114, the list of the 60 municipalities, should be provided at least as supplementary information

R27 Response. We added a supplement with this information.

28. Regarding the health care utilization rates(lines 146-156), what kind of rate do you include: incident rates or count-based (repeated) rates? Also, were they calculated from the overall March 1 through July 31, 2020? Or in a monthly based interval (Monthly rates) over such an overall study period?

R28 Response. The data analysis for incidence rates could allow more than one event per person. Because the COVID activities had a different reference period, we calculated theCOVID-19 case rate (an incidence rate) as a weekly rate. For overall service utilization, we calculated an overall period rate. The notes to each figure indicate where weekly rates were used. See line 198,

29. Why were emergency department visits not considered? Emergency department visits are considered an important indicator of healthcare needs, utilization and accessibility, especially among underserved population groups.

R29 Response. With the paper’s goal of contrasting comprehensive versus safety net services, we focused on types of care to best fit that paradigm, that is consultations rather than emergency room visits.

30. COVID-19 cases and deaths and overall deaths. The authors stated that they have no reason to suspect any widespread or systematic errors in the death data. What about misreporting issues, both underreported and overreported COVID-19 death data?

R30 Response. We added a sentence to distinguish access to servers from accuracy of reporting test results, worded as follows: Our analysis sought to distinguish service access from data quality (see lines 471-472).

31. Please provide the death data period used. For example, was the same data period for healthcare utilization outcomes?

R31 Response. The time period for deaths is April 26 through Nov. 28, 2020 – please see line 216. These dates are shown in the legends to the relevant figures. These vary somewhat by data item based on data availability. Also see R33.

32. Analytical approach, rates per 100,000 population, are those for hospitalization, consultation incident or repeated event rates?

R32 Response. The data are event rates during the study period. It is possible that one person had more than one event (e.g. more than one consultation). The analyses are based on aggregate numbers of events and population rather than person-level data.

33. Is there any reason the chosen death-related period differed from the healthcare utilization period?

R33 Response. Colombia did not begin tracking COVID-19 deaths until April 2020, about one month into the pandemic. Utilization was tracked from the start of the pandemic. For utilization, we did not want to ignore data from the first month of the pandemic, which we would have had to do to obtain perfect consistency.

34. What statistical models or methods did you use to compare the rates of healthcare utilization and deaths across the two compared populations and pre-and post-pandemic periods?

R34 Response. We have expanded on the section on analytic approach. Most comparisons were based on correlations and regressions. Please see lines 240 – 245

Ethical exemption

35. As stated in a previous comment, the country or jurisdiction of the Institutional Research Board (REB) that approved the study needs to be stated. Also, why any Colombian-based REB was involved?

R35 Response. As Brandeis University was the lead recipient for the ELRHA funding and led the utilization analysis for Columbia University funding, Brandeis had ultimate responsibility to sponsors for these analyses. This responsibility required approval by the Brandeis ethics board. As the study was exempted, the Brandeis IRB did not require approval from a Colombian ethics committee. Please see the Ethical exemption section beginning on line 262.

**Results**

36. Lines 223-224. Please specify the two periods that you segmented and compared, as it is not clear from the provided information.

R36 Response. We added an additional document of Supplementary Information and refer to section S2 of it here.

37. Line 232. What are those similar factors? Shared area-based factors? Individual factors?

R37 Response. For clarity, we used the word “municipalities” for area-level factors and changed “factors” to “multiples” in the next sentence referring to numbers. Please see lines 284-285

38. Lines 243 and 245. Where did those results remain after being adjusted by age? As it is likely that there are other factors beyond age that may explain such differences, such as COVID-19 exposure-related factors, health status, sex and gender.

R38 Response. We shifted the explanation about age adjustment to discussion but noted in the limitations our limited ability to control for demographic factors and our inability to examine or control for other socio-economic or exposure factors – please see the second limitation starting at line 534 with the limitations section.

39. Lines 285 and 286. Also, they may be likely due to the prioritization of COVID-19-related healthcare over traditional services for non-COVID-19 health issues as a strategy to reduce the healthcare system collapse. This may also be related to the healthcare staffing shortage. Again, however, the interpretations can be moved to the discussion rather than have them in the result.

R39 Response. Our discussion now acknowledges key barriers around access to health services (see line 337). In response to the comment on health system collapse, Supplementary Information S3 and S4 show differential changes in utilization for various diagnostic groups.

40. Line 294, (by 42% (1.00-0.58)) is the within bracket figures a confidence interval or interquartile range?

R40 Response. We replaced the minus sign by the word “minus” to correct the misunderstanding. Please see lines 340 and 349.

41. 41. Line 313 to 314. Again, the interpretation of findings should be left for discussion.

R41 Response. This interpretation about fear of exposure to COVID-19 from a visit to a health facility was moved.to around line 354.

42. Is there any correlation between consultation and hospitalization for each studied population group? For example, where are the rates of consultation correlated to the hospitalization rates in the Colombian and Venezuelan populations?

R42 Response. We appreciated this useful suggestion. We added a sentence to the methods and added Figure 6 (panels A and B) and associated text to explain the results – a discussion of Figure 6 begins at line 386.

43. Finally, is there any reason biological sex or gender, as well as other socio-economic factors such as education level, and health status, were not considered in the analyses?

R43 Response. We explained in the ln the limitations that our access to RIS data allowed only aggregate tabulations – see line 524

# Discussion

44. Lines 336 to 340, despite age, can be one of the explanations. It is likely that biological sex and gender also influence the findings. The authors stated genocide as a potential cause of Venezuelan migration, but I would be very cautious of using that word without evidence. Yet, it is important to recognize that Venezuelans have suffered different and systematic human rights violations. However, I don’t think the study findings and type of study can be used to make the genocide assertion.

R44 Response. We removed the mention of genocide.

45. Also, regarding the healthy immigrant effect, it is important to highlight that such an effect could reduce with time when migrants assimilate local environmental or behavioural factors. Resilience can be another explanatory factor. <https://link.springer.com/article/10.1007/s10903-021-01313-5>

R45 Response. We appreciate this citation and have added the resilience factor and expanded the discussion of the selection effect. We incorporated the Elshahat publication – it is now reference 29 and is used at line 408.

46. Lines 358, there is likely a huge misreporting effect among Venezuelans due to different factors, such as slow access to technologies (PC, Smartphone) to access healthcare services virtually, which were common during the pandemic. Also, it is likely that due to fear of being discriminated against and stigmatized as a potential virus spreader and as a high utilizer of healthcare services and social services, and the high criminalization they have experienced (https://[www.crisisgroup.org/latin-america-](http://www.crisisgroup.org/latin-america-) caribbean/andes/colombia-venezuela/hard-times-safe-haven-protecting-venezuelan ; https://[www.unhcr.org/en-ie/61f40a914.pdf,](http://www.unhcr.org/en-ie/61f40a914.pdf)

https://globalizationandhealth.biomedcentral.com/articles/10.1186/s12992-020-00655-3

), Venezuelans may have used fewer healthcare services and reported fewer COVID-19 symptoms. Also, in the Colombian context, people still have socio-economic and limited access to COVID-19 testing; therefore, underreporting is expected.

R46 Response. We added a section on discrimination. In it, we included a reference to the UNHCR Somos Panos report (reference 41 by Durán et al.) and the Crisis Group Report (based on the “Hard Times” reference 42.) Please see lines 581-585

47. Line 366, the assertion “while Colombians, knowing that they had good access to comprehensive care” are not backed for any reference. As a Colombian, I rather believe that many Colombians, especially those in the middle or lower social class, have a lot of complex and interrelated socio-economic unmet needs; therefore, they cannot afford not to go out and look for survival resources, including working even without appropriate personal protection equipment, exposing them to a higher risk of contracting COVID-19. https://jech.bmj.com/content/76/2/116 https://[www.ncbi.nlm.nih.gov/pmc/articles/PMC7934198/](http://www.ncbi.nlm.nih.gov/pmc/articles/PMC7934198/) https://[www.ncbi.nlm.nih.gov/pmc/articles/PMC8940255/](http://www.ncbi.nlm.nih.gov/pmc/articles/PMC8940255/)

R47 Response. Thanks for these citations. The text has been revised. Also, the study by Cifuentes (reference 33) was cited around lines 419 and 468. The study by Prada et al (reference 38) is cited around line 509.and Gómez-Restrepo (reference 34) is cited around line 454.

48. Lines 377-379. I think the study‘s data do not support this statement. Despite the Colombian government's efforts to strengthen the health services resources during the pandemic pick, people still experienced limited access to health services for many non- covid-19 problems. https://[www.frontiersin.org/articles/10.3389/fpubh.2022.896318/full](http://www.frontiersin.org/articles/10.3389/fpubh.2022.896318/full) The Colombian government also had serious limitations in implementing key health initiatives, such as early vaccination, which could reduce the severity of COVID-19 cases and associated death. <https://pubmed.ncbi.nlm.nih.gov/35340774/>

R48 Response. The authors thank the reviewer for bringing these two papers to our attention. We cited both and made revisions to the discussion at the lines the reviewer highlighted and under “recent policy changes” (around line 509, Prada et al,. reference 38. The Prada paper, though having different link, was also cited in R47.

49. Among the recommendations, strategies to tackle discrimination and stigma against Venezuelans in the Colombian context should be considered at the individual, population and structural levels, including healthcare settings, to decrease potential health and social inequalities in Venezuelans in Colombia.

R49 Response. We agree. We noted the problem of discrimination and stigma around line 440 and added a section on recommended policy initiatives against discrimination stigma beginning at line 581

50. “With international support, Colombia’s well-designed national health system could address many of these needs. However, this statement is so general. What do you mean by Colombia’s well-designed national health system?

R50 Response. We re-worded the section to clarify our focus on the subsidized regime in Colombia’s national health insurance. Please see lines 497-499..

51. A limitations section needs to be included.

R51 Response. This has been added starting on line 510.
